# Reduced body weight at weaning followed by increased post-weaning growth rate interacts with part-per-trillion fetal serum concentrations of bisphenol A (BPA) to impair glucose tolerance in male mice

**DOI:** 10.1371/journal.pone.0208846

**Published:** 2018-12-17

**Authors:** Julia A. Taylor, Jennifer M. Sommerfeld-Sager, Chun-Xia Meng, Susan C. Nagel, Toshi Shioda, Frederick S. vom Saal

**Affiliations:** 1 Division of Biological Sciences, University of Missouri-Columbia, Columbia, Missouri, United States of America; 2 Department of Obstetrics, Gynecology and Women's Health, University of Missouri-Columbia, Columbia, Missouri, United States of America; 3 Massachusetts General Hospital Center for Cancer Research, Charlestown, Massachusetts, United States of America; The University of Manchester, UNITED KINGDOM

## Abstract

There is evidence from longitudinal studies that being light at birth and weaning is associated with subsequent rapid weight gain in infants. This is referred to as “centile crossing”, which can lead to increased risk of lifetime obesity, glucose dysregulation and type 2 diabetes. Here, pregnant CD-1 mice were hemi-ovariectomized so that the entire litter was contained in one uterine horn to increase variability in fetal growth rate. Pregnant females were implanted on gestation day (GD) 9 with a Silastic capsule containing 6, 60 or 600 μg bisphenol A (BPA). On GD 18 the mean fetal serum unconjugated BPA concentrations were 17, 177 and 1858 pg/ml, respectively. Capsules were not removed, to avoid maternal stress, and were predicted to release BPA for at least 3 weeks. Body weight at weaning was strongly negatively correlated with post-weaning weight gain in both control and BPA-treated male mice, consistent with human data; female offspring were excluded, avoiding complications associated with postpubertal estrogens. Within each treatment group, male offspring were sorted into tertiles based on relative weight gain during the two weeks after weaning, designated as having Rapid (R), Medium (M) or Slow (S) growth rate. BPA exposure was associated with altered growth rate between weaning and postnatal week 12 (young adulthood), when a low-dose (20 mg/kg, i.p.) glucose tolerance test (GTT) was performed. We found altered glucose regulation in response to all doses of BPA. However, glucose tolerance was only significantly impaired (blood glucose levels were elevated) compared to controls in males in the rapid post-weaning growth group exposed perinatally to BPA. We conclude that male mice that are light at weaning, but then experience rapid catch-up growth immediately after weaning, represent a sensitive sub-population that is vulnerable to the metabolic disrupting effects of very low pg/ml fetal serum concentrations of BPA.

## Introduction

Decades ago type 2 diabetes was considered an adult-onset disease. Adolescents and even children have experienced significant increases in both obesity and type 2 diabetes over short periods of time [1, 2]. Children who become obese are likely to remain obese and develop insulin resistance and ultimately type 2 diabetes as adults, resulting in extremely high health care costs associated with treating the many components of metabolic disease [1]: cardiovascular disease, hypertension, dyslipidemia, liver and gallbladder disease, insulin resistance, hyperglycemia and type 2 diabetes [3]. The consequence of the increase in disease burden associated with diabetes and obesity is that children born in the 21^st^ century are not predicted to have the life expectancy of their parents [4].

The risk for obesity and other components of metabolic disease is highest in two subgroups of infants: those that are born heavy and remain heavy, and also those that are light at birth and then experience accelerated early childhood growth [5], a phenomenon known as “centile crossing”. Growth velocity during the first few years of life is particularly important as a predictor of adult obesity in people [6], which is a predictor of glucose dysregulation leading to insulin resistance and type 2 diabetes [3]. Children who experience intrauterine growth restriction (IUGR) and then show rapid growth during infancy into childhood (crossing two growth percentiles) show an increased risk for obesity and subsequent co-morbidities [7, 8]. The combination of low birth weight and accelerated postnatal growth impacts glucose tolerance at age 7, when compared with light at birth babies that did not experience the accelerated growth [9]. Determining the factors that contribute to glucose dysregulation and other co-morbidities of metabolic syndrome has thus become a major public health issue [3].

The change in incidence of obesity and type 2 diabetes that occurred during the 1990s and the first decade of the 21^st^ century have been considered by most physicians to be due to changes in diet and exercise habits, but there is now considerable evidence that other factors may also be involved [3]. Within the last couple of decades considerable research has focused on a class of environmental chemicals that can disrupt the normal functioning of the endocrine system, referred to as endocrine disrupting chemicals (EDCs). EDCs are used in agriculture (pesticides), household products (building materials, plastics, cosmetics, cleaning materials), and are found in food, beverages, water and air [10]. The EDCs that can disrupt metabolic function and that are implicated in the etiology of metabolic disease are referred to as “metabolic disrupting chemicals” [3]. One of the highest production volume EDCs, that has been shown to be a metabolic disruptor, is bisphenol A (BPA), a synthetic monomer used to make polycarbonate plastic and resins, and an additive in many consumer products. BPA is an estrogen receptor agonist but also interacts with other hormone receptors [11].

We previously reported that prenatal exposure of male CD-1 mouse fetuses to low but not high doses of BPA via maternal oral treatment resulted in an increase in adipocyte size and number, altered serum hormones that impact glucose regulation, and impaired glucose tolerance [12]. Others have found similar effects of prenatal BPA exposure on metabolic outcomes in rodents that include reduced glucose tolerance and increased insulin resistance, increased body weight and altered pancreatic islet function [13–19]. There are reported associations between BPA exposure and impaired fetal growth and metabolic outcomes in humans [20, 21], as well as studies relating BPA exposure to obesity in children [22] and altered insulin secretion in men and women. We have also previously shown increased pre-weaning growth rate in CF-1 mice exposed to BPA during gestation [23]. These effects of BPA on postnatal growth and metabolic outcomes in rodents thus appear to mirror the effects of early accelerated growth rate (due to unknown variables) on metabolic outcomes in humans, and suggest a potential model for examining the interaction of growth rate during early life and BPA in the etiology of glucose intolerance.

Here we examined the relationship between body weight at weaning and post-weaning growth rate, and determined the impact of these factors on the effect of fetal/neonatal exposure to three low doses of BPA on glucose tolerance. We utilized the crowded uterine horn model to increase variation in fetal growth rate; this model was proposed by McLaren in 1960 [24], and generates siblings that range from growth-restricted to macrosomic due to differences in placental blood flow, based on their location in the uterus [25, 26]. The effect of crowding fetuses in a single uterine horn on blood flow to fetuses at the ends vs. the middle of the uterine horn is shown in S1 Fig in Supporting Information [27]. We administered BPA to pregnant and lactating mice using Silastic capsules for continuous release to best model continuous human exposure to BPA [28], and measured the resulting concentrations of unconjugated (bioactive) BPA and BPA metabolites in maternal and fetal serum. We report adverse effects of fetal/neonatal exposure to low (pg/ml) serum concentrations of unconjugated BPA on glucose tolerance in male CD-1 mice at 3 months of age. The greatest effects occurred in male mice that were in the lowest tertile for body weight at weaning and then exhibited the highest rate of growth during the first two weeks after weaning. We thus identify here a relationship between weaning weight (that is correlated with birth weight), post-weaning growth rate, and exposure to very low levels of BPA during development, on adult glucose tolerance in male mice. We focused on males in this study because females typically reach puberty during this post-weaning period of rapid growth, and the rising estrogen levels in females impact feeding behavior, pancreatic function [29] and weight gain and thus complicate interpretation of results.

## Methods

### Chemicals and standards

Bisphenol A (≥99% pure) was obtained from Aldrich (St. Louis, MO). Authentic tritiated BPA (10.8 Ci/mmol) was obtained from Moravek Biochemicals (Brea, CA). Tocopherol-stripped corn oil and Ecolite(+) scintillation fluid were obtained from MP Biomedical (Santa Ana, CA). Silastic tubing (Dow Corning 2415569, 0.062 in I.D. x 0.125 in O.D.) was obtained from Fisher Scientific (Waltham, MA). ß-glucuronidase (type H-1) was obtained from Sigma Aldrich (St. Louis, MO). Other reagents were obtained from Fisher Scientific (Waltham, MA) and were HPLC grade where available.

For BPA capsules, BPA was dissolved in tocopherol-stripped corn oil at 0 (oil vehicle alone), 6, 60 or 600 μg BPA in 20 μl oil, and these solutions were used to fill Silastic capsules (20 μl/capsule, 1 cm between the capped ends).

### Animals

Animal care conformed to the NIH Guide, and The Animal Care and Use Committee at the University of Missouri approved all procedures prior to conducting the study.

Ten-week-old virgin female CD-1 mice were purchased from Charles River Laboratories (Raleigh, NC) and were housed in polypropylene cages with corncob bedding in a temperature- and humidity-controlled facility on a 12L:12D cycle. We have examined water (purified by RO and carbon filtration and supplied in glass bottles *ad libitum*) for BPA contamination, and levels are below the limit of detection for BPA in water in our LC-MSMS assay (limit of detection, 0.01 ng/mL for this assay of BPA in water).

All females were hemi-ovariectomized, with removal of the left ovary; this procedure increases the variance in fetal body weight by crowding one litter into a single uterine horn, because the normal number of ova from both ovaries is ovulated by the one remaining ovary [24, 27]. One week after surgery females were paired with an individually housed male, transferred to Purina 5008 pregnancy diet (6.5% fat), and allowed to produce a single litter. The first litter was removed at parturition, and the female was allowed to mate a second time: a female with a new litter typically enters postpartum estrus approximately 24 hr after parturition, and thus the timing of the birth of the first litter was used to predict the gestation stage of the second litter without having to time mate and handle the females to determine if there was a vaginal plug; this procedure was used to reduce maternal stress and the associated ~20–25% termination of pregnancy associated with examining females for vaginal plugs. The estimated day of mating was designated gestation day (GD) 0. This second mating step was also performed because we have observed that a CD-1 female’s first litter is smaller than the subsequent litter [30], and we wanted to maximize the crowded uterine horn effect.

Pregnant females were randomly assigned to BPA or vehicle treatment. On the estimated GD9 of the second litter, females were implanted with a subcutaneous Silastic capsule containing 0, 6, 60 or 600 μg of BPA in tocopherol-stripped corn oil (MP Biomedical). Capsules were not subsequently removed, to avoid maternal stress, and were predicted to release BPA over a period of at least 3 weeks (Even and vom Saal, 1991). We started exposure at GD 9 to more easily relate this study to our past experiments that have used this approach to identify critical developmental periods for the effects of BPA exposure on various outcomes including growth and glucose regulation. On GD17 pregnant females were singly housed and allowed to deliver naturally on GD19, which occurred for all females. Pup weight and sex were recorded on postnatal day (PND) 2, but otherwise the litters were left undisturbed until weaning on PND21. Litters with less than 9 pups, or litters with only one male or one female, were excluded from the remainder of the study. This resulted in final litter numbers of 5, 9, 8 and 9 for 0 (controls), 6, 60 and 600 μg BPA capsules respectively; these four exposure groups are later referred to as Control, BPA-17, BPA-177 and BPA-1858, based on the average fetal serum BPA concentrations (pg/ml) on GD18 (see Table 1). At weaning the animals were ear-clipped for identification, weighed and rehoused at 2–4 of the same sex per cage by treatment. At this time the animals were transitioned to the slightly lower fat Purina 5001 maintenance diet (4.5% fat); both Purina 5008 and 5001 are soy-based diets. After weaning, food consumption was recorded three times a week between weeks 3–5, for a total of 6 measures per cage. Body weight was recorded at 3, 4 and 5 weeks, and at less frequent intervals thereafter.

**Table 1:** Serum ^3^H-BPA concentrations.

		———Maternal serum———			——Fetal/Neonatal serum—-		
Capsule Dose	Fetal Age	Unconjugated ^3^H-BPA	Conjugated ^3^H-BPA	N	Unconjugated ^3^H-BPA	Conjugated ^3^H-BPA	N
6 μg	GD14	18.1 ± 4.7	85.4 ± 48.1	3			
	GD18	27.4 ± 3.4	62.7 ± 43.1	4	16.6 ± 1.2	543.9 ± 50.0	3
	PND2	15.4 ± 11.4	131.7 ± 29.5	2	2.7 ± 1.6	28 ± 22.6	2
60 μg	GD14	278 ± 15.5	800.2 ± 174.2	3			
	GD18	295.5 ± 32.4	357.5 ± 107.4	4	176.5 ± 23.3	4778.5 ± 461.7	4
	PND2	183.2 ± 134.7	1886.3 ± 1148.9	2	85.9 ± 34.5	499 ± 398.5	2
600 μg	GD14	2266.3 ± 1009.1	6149.2 ± 2820.7	3			
	GD18	2832.3 ± 204.3	4031.5 ± 2032.9	4	1857.6 ± 135.3	34456.6 ± 3629.0	4
	PND2	2904.8 ± 781.2	27435.2 ± 13663.1	2	910.5 ± 451	7533 ± 1457.5	2

Mean (±SEM) unconjugated and conjugated serum ^3^H BPA concentrations (pg/ml) in maternal serum and fetal or PND2 pup serum in CD-1 mice exposed via the mother to Silastic capsules containing 6, 60 or 600 μg BPA, measured on GD14 (maternal serum only), GD18 and PND2. N = number of individual maternal samples and litters (pooled fetal/neonatal samples) assayed.

### Separation of treatment groups by weight-gain tertile

Two weeks after all animals were weaned, they were sorted *within* treatment groups by percent weight gain during the two weeks after weaning (weeks 3–5) without regard to litter identity, since the crowded uterine horn model (S1 Fig) is specifically focused on comparing light, median and heavy-at-birth siblings; thus, siblings were typically represented in the different tertiles. For each individual treatment group, growth rate tertiles were established using the 33^rd^ and 67^th^ percentiles of weight gain as cutoff/transition points, and throughout the study we identify animals in relative terms as having rapid (R), medium (M) or slow (S) growth rate during the 2-week period after weaning. Due to the method used to assign tertiles, there were approximately equal numbers of animals in the R,M and S tertiles within each BPA treatment group: numbers of animals in the R, M, and S categories by treatment were: Control n = 9, 10 and 9; BPA-17 n = 22, 22 and 22; BPA-177 n = 10, 10 and 10; and BPA-1858 n = 18, 17 and 18 respectively.

### Serum BPA measurements

Initial studies were conducted to determine serum BPA concentrations resulting from the Silastic capsule treatment protocol. For these studies we mixed ^3^H-BPA (10.8 Ci/mmol) with non-radioactive BPA to give solutions that contained 6, 60 or 600 μg BPA per 20 μl oil, and used these mixed solutions to fill the capsules. Samples of each solution were taken to confirm the radioactivity used at each concentration, from which the specific activities (Ci/mmol) were calculated. We targeted the same amount of radioactivity in each dosing solution, and thus the specific activities decreased approximately 10-fold as the concentration of unlabeled BPA increased across our dose range, with the lowest specific activity in the highest dosing solution. The resulting limits of detection for BPA in serum were 3.8, 40.9 and 427.8 pg/ml for the 6, 60 and 600 μg capsule exposures respectively. Radioactive BPA was used for improved sensitivity, particularly for fetal serum BPA concentrations where sample volumes were low and not sufficient for measuring unconjugated (bioactive) BPA in the parts per trillion range by LC/MSMS. Note that the small amount of serum collected from mouse fetuses and the consequent decision to use ^3^H-BPA precluded measurement of serum BPA concentrations in the control fetuses.

Randomly-selected pregnant females were implanted on GD11 and left group-housed until GD17. Animals were euthanized under CO_2_ on GD14, GD18 or postnatal day (PND) 2. Maternal blood and fetal or pup blood (pooled by litter) were collected and left on ice to clot. Serum was separated by centrifugation at 4°C and then stored at -20°C prior to assay. For serum ^3^H-BPA quantification, aliquots of serum were extracted twice with methyl tert-butyl ether (MtBE), either before or after deconjugation with beta-glucuronidase to determine unconjugated and conjugated serum ^3^H-BPA concentrations. Recovery of BPA by MtBE extraction was determined in parallel-run control serum samples containing known amounts of ^3^H-BPA, and averaged 87%. Deconjugation efficiency of authentic BPA glucuronide was determined separately in another study [31] and calculated to be 90% using our published method. Extracted ^3^H-BPA in serum was quantified by scintillation counting, and calculated amounts were corrected for assay efficiency as described previously [32].

### Glucose tolerance test

A glucose tolerance test was conducted at 3 months of age. Mice were fasted for 4 hours with full access to water (preliminary studies showed that typical overnight fasting resulted in a significant loss of body fat in mice and thus was not used here). Glucose (20 mg/kg BW) was administered by i.p. injection in 0.9% saline, with the volume (~160 μL) adjusted for body weight to maintain a constant dose. Tail blood was collected by tail vein nicks before glucose challenge and again at 30, 60 and 120 min after injection. Blood glucose was measured using an Accu-Chek Aviva plus glucometer (Roche, Indianapolis, IN). The area under the curve (AUC) for the blood glucose levels over the 120 min monitored after injection was calculated using the linear trapezoidal rule. The percent change in blood glucose from baseline was also calculated.

### Statistical analysis

Data were analyzed by ANOVA, GLM procedure using SAS version 9.0. The main effect variables were treatment, week 3–5 rate of growth tertile, and the both the area under the concentration-time curve (AUC) and repeated measures in the GTT study. Planned comparisons were made using the LSMeans test. Statistical significance was set at P < 0.05.

## Results

### Serum BPA measurements

Unconjugated ^3^H-BPA concentrations in fetal serum (GD 18) were linear with dose (Table 1). In CD-1 mice exposed to capsules containing 6, 60 or 600 μg BPA spiked with ^3^H-BPA, mean fetal serum concentrations of unconjugated BPA on GD 18 were 16.6, 176.5 and 1857.6 pg/ml, respectively (in the figures these values are rounded to 17, 177 and 1858 pg/ml respectively). We also measured unconjugated and conjugated BPA in the pups on PND2, the presence of which may have been due to residual BPA from the prenatal, transplacental exposure and/or transfer from mother to pup via nursing, since the capsules were not removed from the mothers at parturition to avoid the stress associated with surgery. The four capsule treatment groups (0, 6, 60 and 600 μg BPA/capsule) are referred to hereafter in terms of the (rounded) GD 18 fetal serum BPA concentrations, as Ctrl, BPA-17, BPA-177 and BPA-1858 exposure groups, respectively.

As indicated in the Methods, we used ^3^H-BPA to aid in the measurements of fetal serum BPA concentrations because of low sample volumes sensitivity issues. However, the drawback to this approach is the lack of a measured serum BPA concentration in control (non-treated) fetal serum. Thus, the concentrations measured in BPA-treated animals are in addition to any background. However, assays of our untreated adult mouse serum in other experiments using LCMS-MS have not detected BPA, and here we controlled for background exposures: cages are made of BPA-FREE polypropylene, and past screenings of drinking water (purified by reverse osmosis and carbon filtration) and feed have been negative. In addition, the ability to detect effects of the lowest BPA exposure in this experiment (see later in Results) suggests an extremely low or even zero background. However, because of this limitation, although the BPA-treated animals are identified in the figures by their measured serum ^3^H-BPA concentrations, control animals are identified simply by “C” since their serum was not directly measured due to the use of ^3^H-BPA in BPA-containing capsules.

### Relationship between body weight at weaning and rate of growth during the following two weeks

**Fig 1:**
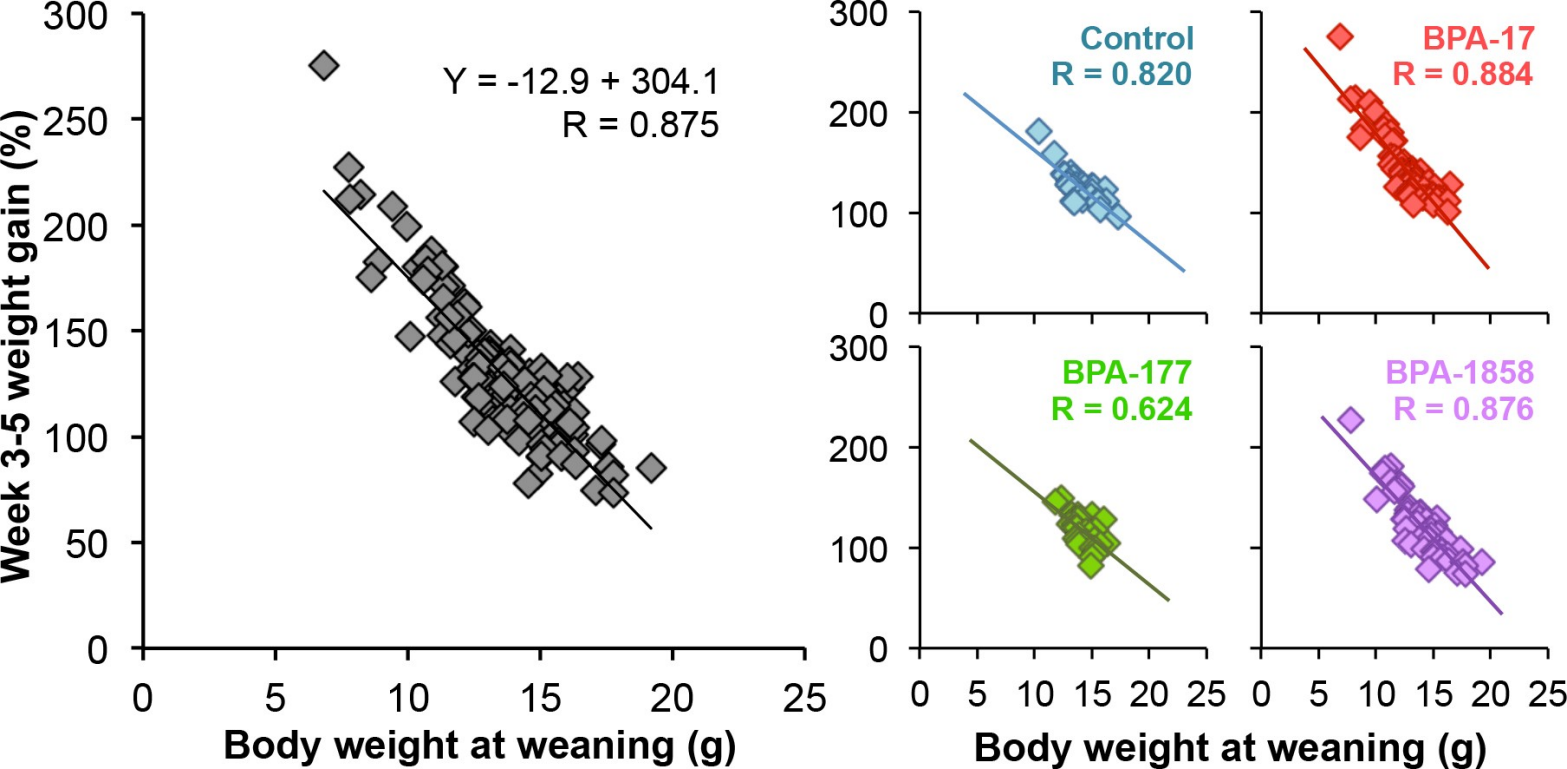
Relationship between weaning weight and post-weaning weight gain between week 3–5 in male mouse offspring. Left: data for all animals without regard to perinatal treatment, and Right: the slopes and correlation coefficients for each individual treatment group.

The relationship between weaning weight and weight gain over the next two weeks (between weaning and early adolescence from PND 21–35) in this study is shown in Fig 1. A strong inverse relationship between weaning weight and post-weaning weight gain was found, either when animals from all treatments were combined (R = 0.875) or when each treatment was examined individually (R = 0.8197, R = 0.8835, R = 0.6242 and R = 0.8756 for Ctrl, BPA-17, BPA-177 and BPA-1858, respectively).

**Fig 2:**
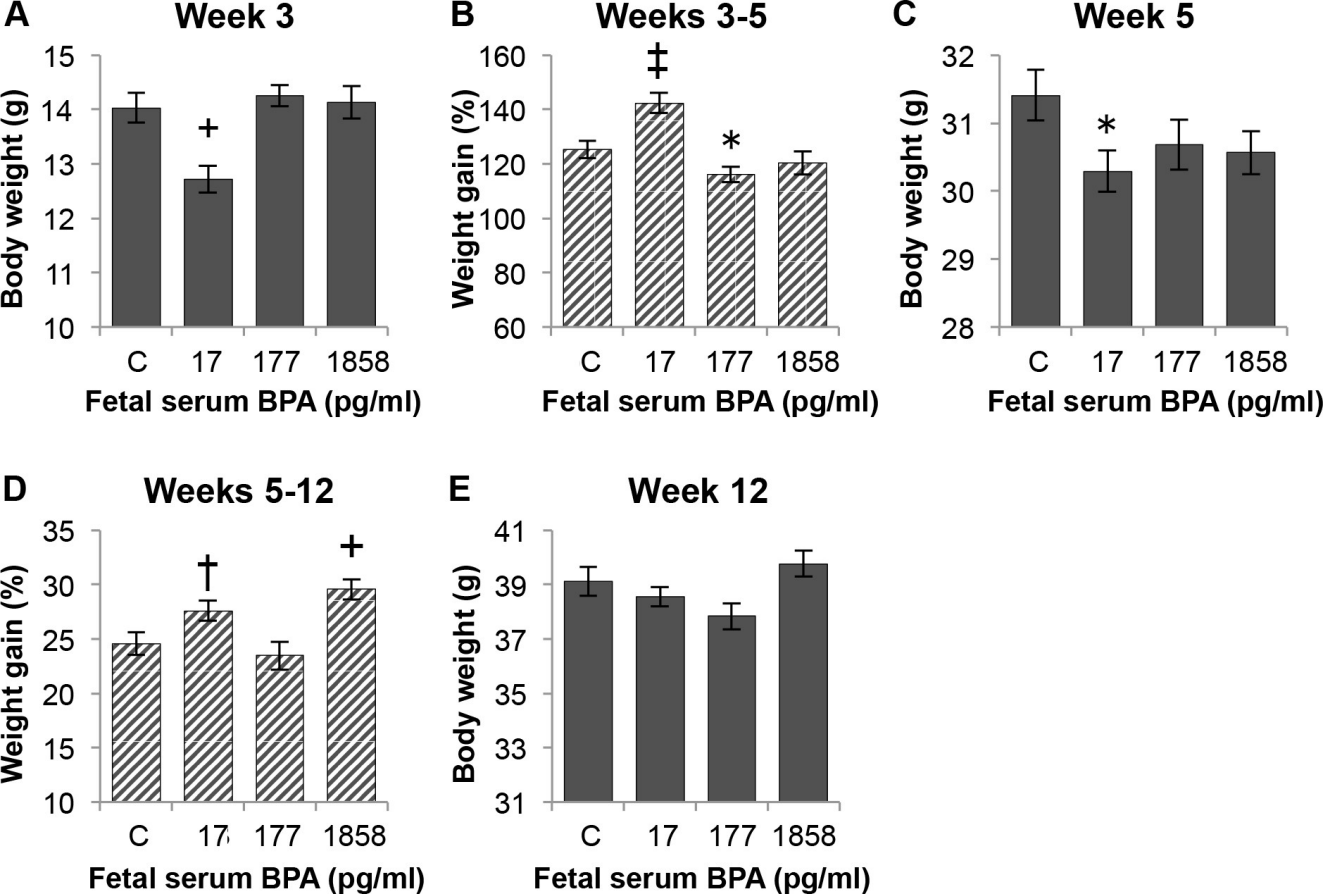
Effect of perinatal serum BPA concentration on weaning weight and post-weaning weight gain. Animals from the three different growth tertiles are combined in each treatment group in this figure. Panel A) body weight of males in each treatment group at weaning on week 3. Panel B) weight gain between weeks 3–5. Panel C) body weight at week 5. Panel D) weight gain between weeks 5–12. Panel E) body weight at week 12 (the BPA-1858 males were significantly heavier than the BPA-17 or BPA-177 males; P < 0.05). Data are mean±SEM. Comparisons of BPA exposure group vs. control group: ^†^P = 0.06, *P<0.05, ^+^P<0.01, ^‡^P<0.001. See also S2 Fig in Supporting Information.

The effect of perinatal BPA dose on body weight at weaning and on post-weaning weight gain is shown in Fig 2. Fig 2A shows the effect of perinatal BPA dose on body weight of males at weaning, with the BPA-17 group significantly lighter than controls (P < 0.01). While the BPA-17 males had a significantly greater percent increase in body weight between week 3–5 relative to controls (Fig 2B), the BPA-17 males were still significantly lighter than controls (P < 0.01) when 5 weeks old (Fig 2C). Between week 5–12 the percent increase in body weight was significantly greater for BPA-1858 males compared to controls (P < 0.01); BPA-17 males also showed a tendency for increased weight gain compared to controls for (P = 0.06) (Fig 2D). The result was that by week 12 there were no significant differences in body weight between animals BPA-exposed males relative to control males (Fig 2E). However, there was a significant treatment effect (P < 0.05) at week 12, and the BPA-1858 males had a significantly greater body weight than either the BPA-17 (P < 0.05) or BPA-177 males (P < 0.01), so there were significant differences in body weight at week 12 due to BPA dose.

**Fig 3:**
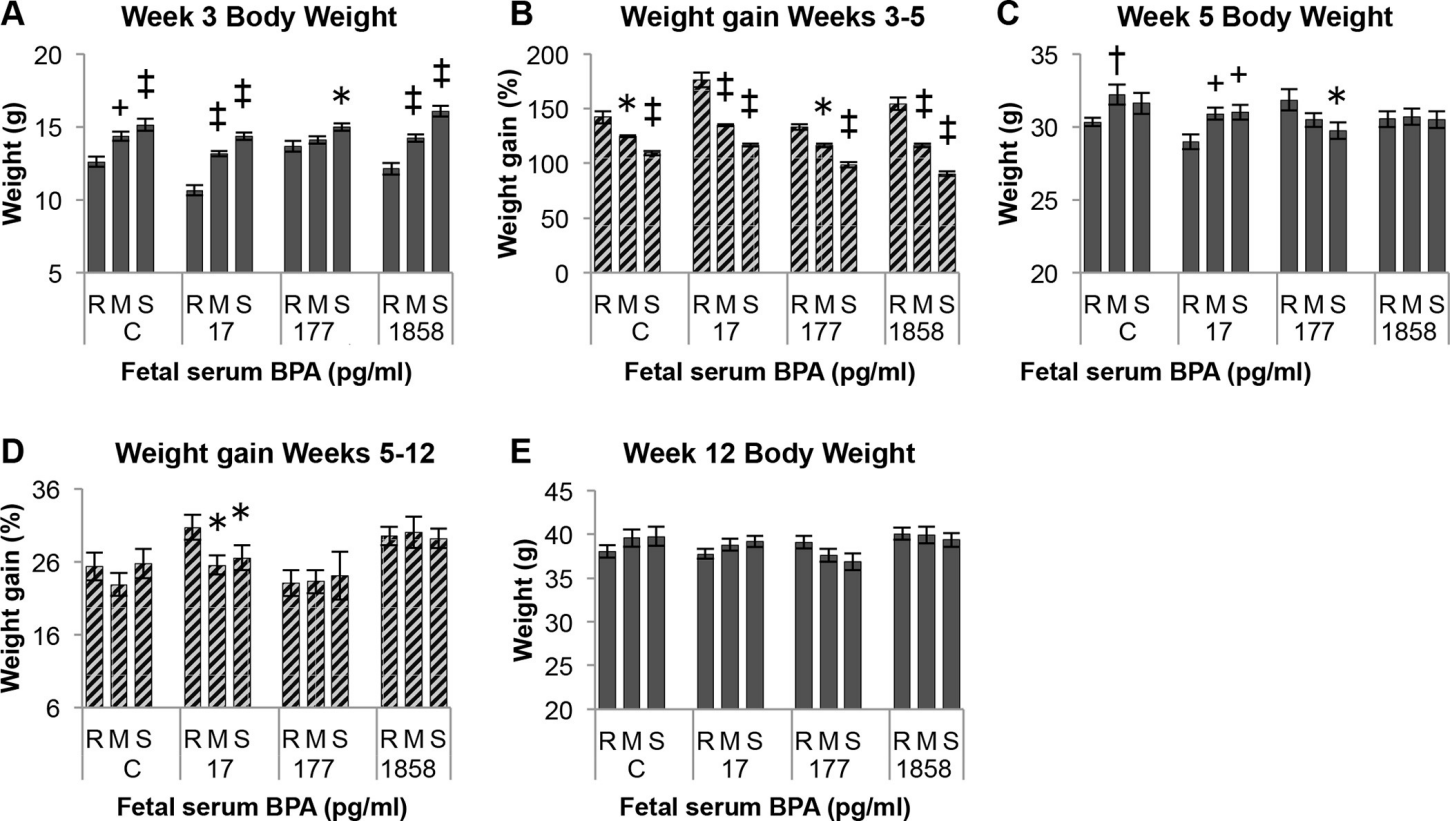
Effect of treatment on body weight and weight gain by growth tertile. Each treatment group is divided into tertiles based on growth rate over weeks 3–5. Data show the effect of fetal BPA serum concentration and post-weaning growth tertile on: Panel A) body weight at weaning on week 3. Panel B) weight gain between weeks 3–5. Panel C) body weight at week 5. Panel D) weight gain between weeks 5–12. Panel E) body weight at week 12. Values are mean±SEM. Comparison of R vs both M and S groups within each dose group: ^†^P = 0.06, *P<0.05, ^+^P<0.01, ^‡^P<0.001.

Fig 3 shows body weight and percent weight gain for the BPA exposure groups divided into week 3–5 growth rate tertiles (rapid, medium, slow rate of growth). For body weight at weaning (Fig 3A), there was a significant interaction between BPA exposure and post-weaning growth rate tertile (P < 0.01); for all exposure groups the lightest animals were in the rapid-growth tertile and the heaviest animals were in the slow growth tertile. For the four exposure groups, the mean week 3 body weights for the R vs. S tertile were: Controls—rapid = 12.6 g vs. slow = 15.1 g; BPA-17 –rapid = 10.7 g vs. slow = 14.4 g, BPA-177 –rapid = 13.7 g vs. slow = 15.0 g, and BPA-1858 –rapid = 12.1 g vs. slow = 16.1 g.

Post-weaning (week 3–5) weight gain shown in Fig 3B was inversely related to body weight at weaning for all exposure groups, with the greatest difference between the rapid and slow growth tertiles occurring in the BPA-17 and BPA-1858 groups. Thus, the BPA-17 rapid growth males were the lightest of any group with a mean body weight at weaning of 10.7 g, and they showed the highest percent growth between week 3–5 of 176.4%. In contrast, the BPA-1858 slow growth tertile males were the heaviest animals at weaning (16.1 g) and had the lowest percent of week 3–5 percent body weight increase of any group (90.2%).

The data in Fig 3C show that the significantly lower body weight relative to controls for BPA-17 males at week 5 (Fig 2C) was due entirely to the BPA-17 rapid growth males remaining significantly lighter than the BPA-17 medium and slow growth males, in spite of the BPA-17 rapid growth males showing the greatest percent increase in body weight between week 3–5. Between week 5–12, however, the BPA-17 rapid growth males showed significantly higher percent increase in body weight relative to BPA-17 medium and slow growth males (Fig 3D), with the result that by week 12 there were no significant differences in body weight between any of the BPA-treatment males and the controls (Fig 3E).

In contrast to the BPA-17 males, at 5 weeks of age the BPA-177 males in the rapid growth tertile were significantly heavier than males in the slow growth tertile (P < 0.05, Fig 3C), although, as with the other BPA exposure groups, there was no difference in body weight based on tertile for BPA-177 males at week 12 (Fig 3E). While the BPA-1858 males showed the same effect of tertile on body weight at 3 weeks and percent increase in body weight between 3–5 weeks as the other BPA exposure groups, the rapid, medium and slow growth groups reached the same body weight by week 5 (Fig 3C), after which there were no differences due to tertile through week 12 (Fig 3E).

### Food Intake

Food intake during the first two weeks after weaning for males in all groups averaged 211 g Purina 5001 feed per kg body weight per day, with higher intake in the first week after weaning (231±3 g/kg body weight/day during week 3–4 vs. 191±4 g/kg body weight/day during week 4–5). There were no significant differences between treatment groups (Table 2).

**Table 2:** Food intake (grams/kg body weight/day).

Exposure group	Weeks 3–4	Weeks 4–5	N
Control	233.6 ± 6.5	182.8 ± 6.6	5
BPA-17	233.2 ± 5.2	184.6 ± 4.5	9
BPA-177	222.9 ± 3.8	199.8 ± 11.5	8
BPA-1858	232.5 ± 5.7	195.7 ± 11.2	9

Food consumption per day during the first two weeks after weaning in male offspring, expressed as grams/ kilogram body weight/day. Food consumption was measured three times a week across weeks 3–4 and weeks 4–5. Males from the same litter were housed in together. Calculations are based on the total weight of food consumed divided by the number of animals per cage, and then adjusted per average kg body weight of the animals. N = the number of litters examined. Values are mean ± SEM.

### Glucose tolerance test

**Fig 4:**
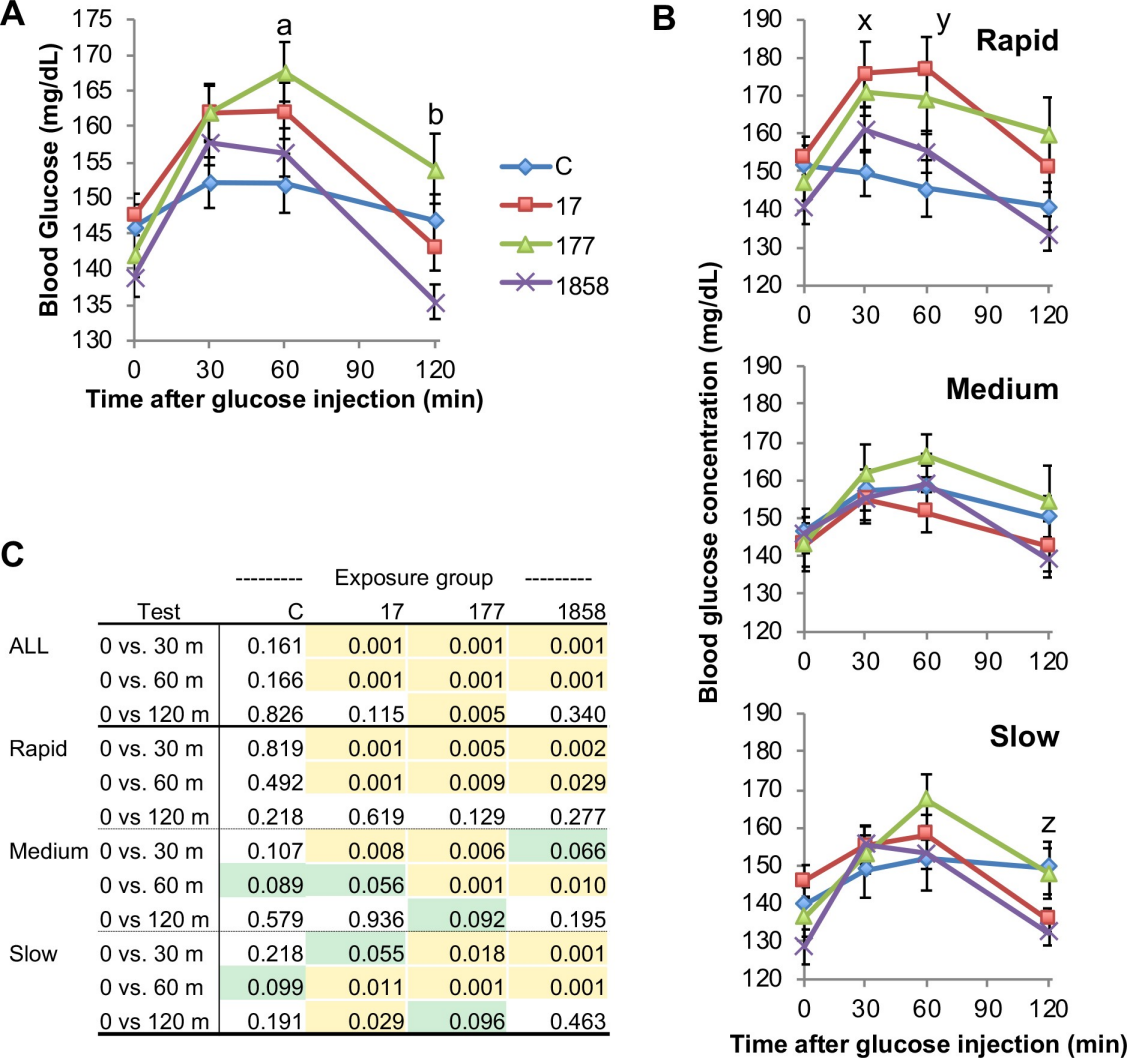
Effect of BPA and post-weaning growth rate on a low-dose glucose tolerance test (GTT). Panel A) Effect of BPA exposure vs. controls (C) without regard to post-weaning growth tertile. Panel B) Effect of BPA exposure within each growth tertile group (rapid, medium, slow). Letters in Panels A and B indicate statistically significant differences between BPA dosed males in comparison to controls at each specific time point. Panel C) The blood glucose response to the glucose injection for males in each separate treatment group at time 30, 60 and 120 min was compared to their time 0 value. P values shaded in yellow for these comparisons are statistically significant relative to time 0 (P < 0.05), while values shaded in green showed a tendency to be different from time 0. Values are mean±SEM. a: Controls vs. BPA-177, P < 0.05; b: Controls vs. BPA-1858, P < 0.05. x: controls vs. BPA 17, P < 0.05. y: controls vs. BPA 17, P < 0.01. z: controls vs BPA-1858, P < 0.05.

Data from the glucose tolerance test are presented in Fig 4 for males in each BPA exposure group without (Fig 4A) and with (Fig 4B) consideration of post-weaning growth tertile.

### Analysis of all males by BPA exposure

An overall ANOVA was conducted on the GTT blood glucose data, which showed a significant interaction (P = 0.002) between perinatal BPA treatment and GTT blood collection time (0, 30, 60, 120 min), with time 0 being pre-glucose administration. We then conducted planned comparisons using the LSMeans test. In Fig 4A (which disregards growth tertile) control males showed no significant change relative to baseline (Time 0) in blood glucose at any time point during the 2 hr after the low-dose (20 mg/kg) glucose injection (P > 0.1). In contrast, relative to their baseline value, the BPA-17 males had significantly elevated blood glucose at 30 and 60 min (P < 0.001) but not at 120 min (P > 0.1), by which time they had returned to baseline. Relative to their baseline value the BPA-177 males had significantly elevated blood glucose at 30, 60 and 120 min (P < 0.005), so even at 120 min after the glucose injection, these males still had significantly elevated blood glucose, indicative of glucose intolerance. Finally, relative to baseline, the BPA-1858 males had significantly elevated blood glucose at 30 and 60 min after glucose injection (P < 0.0001), but not at 120 min (P > 0.1).

Next, we conducted planned comparisons to examine whether blood glucose in males in each BPA-exposure group (disregarding growth tertile) differed from controls at the separate time points examined in the GTT study (Fig 4A). There were no statistically significant differences between controls and males in any BPA-exposure group at baseline (Time 0) or at 30 min, although there was a tendency for the BPA-17 males to have elevated blood glucose relative to controls (P = 0.08), which was also the case at 60 min (P = 0.07). However, the BPA-177 males had significantly elevated blood glucose compared to controls at 60 min (P < 0.05). At 120-min after the glucose injection, the BPA-17 and BPA-177 males did not differ relative to controls. However, the high-dose BPA-1858 males, that had a somewhat lower (not statistically significant) baseline blood glucose level than controls, had significantly lower blood glucose than controls at 120 min (P < 0.05), suggesting that the control of blood glucose levels in the BPA-1858 males differed from controls.

### Analysis of males by BPA exposure group within post-weaning growth tertiles

When separated into the 3 growth tertiles (rapid, medium and slow), the effect of BPA on the response to a glucose injection was shown to have the greatest impact in the rapid-growth males (Fig 4B).

The ANOVA conducted just on males in the rapid growth tertile (Fig 4B) showed a tendency for an effects of BPA treatment (P = 0.06) and a significant effect of Time (P < 0.0001). The rapid-growth controls showed no significant response to the glucose injection. However, relative to their baseline glucose levels, BPA-17, BPA-177 and BPA-1858 males all had statistically significant increases in blood glucose at 30 and 60 min (P values ranged from < 0.05 to < .0001). In contrast, none of the BPA-treatment groups differed from their baseline level and 120 min (P > 0.1). When treatment groups were compared at each individual time point (significant differences are indicated by letters in Fig 4B), there were no significant differences at baseline, but the BPA-17 males differed significantly from controls at 30 min (P < 0.05) and 60 min (P < 0.01) but not at 120 min (P > 0.1). The BPA-177 males tended to differ from controls at 30 min (P = 0.09) and 60 min (P = 0.06), but not at 120 min (P > 0.1). The rapid-growth BPA 1858 males did not differ from controls at any time point.

The ANOVA conducted just on males in the medium growth tertile showed no significant effect of treatment (P = 0.1), a significant effect of Time (P < 0.0001), and no significant interaction between treatment and time (P > 0.1). The medium-growth controls showed no significant response relative to their baseline level following the glucose injection (Fig 4B). In contrast, the BPA-17 medium-growth males had significantly elevated blood glucose at 30 min (P < 0.01) and tended to be elevated at 60 min (P = 0.056), but by 120 min had returned to their baseline level. The BPA-177 males showed a similar response, with elevated blood glucose at both 30 min and 60 min (P < 0.005), and they tended to be elevated relative to baseline at 120 min (P = 0.092). When treatment groups were compared at each individual time point, males in the BPA-treatment groups did not differ from controls at any time point (P > 0.1).

The ANOVA conducted just on males in the slow growth tertile (Fig 4B) showed no significant effect of treatment (P > 0.1), a significant effect of Time (P < 0.0001), and also a significant interaction between treatment and time (P < 0.05). The slow-growth controls showed no significant response relative to their baseline level following the glucose injection (P ≥ 0.1). In contrast, the BPA-17 slow-growth males tended to have elevated blood glucose at 30 min (P = 0.055) and had significantly elevated blood glucose at 60 min (P = 0.01), and by 120 min they had returned to significantly lower blood glucose than their baseline level (P < 0.05). The BPA-177 slow-growth males showed elevated blood glucose at 30 min (P < 0.05) and 60 min (P < 0.001), and they tended to be elevated relative to baseline at 120 min (P = 0.096). The slow-growth BPA-1858 males had the lowest baseline blood glucose levels, and had significantly elevated blood glucose levels relative to baseline at 30 min and 60 min (P < 0.0001), but by 120 min they had returned to baseline levels. When slow-growth treatment groups were compared at each individual time point, males in the BPA-treatment groups did not differ from controls at the 30 min and 60 min time points (P > 0.1). However, at 120 min the BPA-1858 males had significantly lower blood glucose levels relative to controls (P < 0.05).

In summary, an interesting aspect of the results is that regardless of growth rate, none of the control animals showed a significant change in blood glucose during the 120 minutes after the low-dose glucose injection. However, significant responses (elevated blood glucose levels) were seen at post-injection time 30 and 60 min in all BPA-exposed groups whether growth tertile was ignored or included in the analysis (Fig 4C), with the difference relative to baseline being statistically significant (P < 0.05) or showing a tendency to be different (P < 0.1). These findings show that developmental exposure to BPA impaired the ability of adult male mice to control blood glucose levels in response to a low-dose glucose challenge.

### Analysis of area under the glucose concentration time curve (AUC)

**Fig 5:**
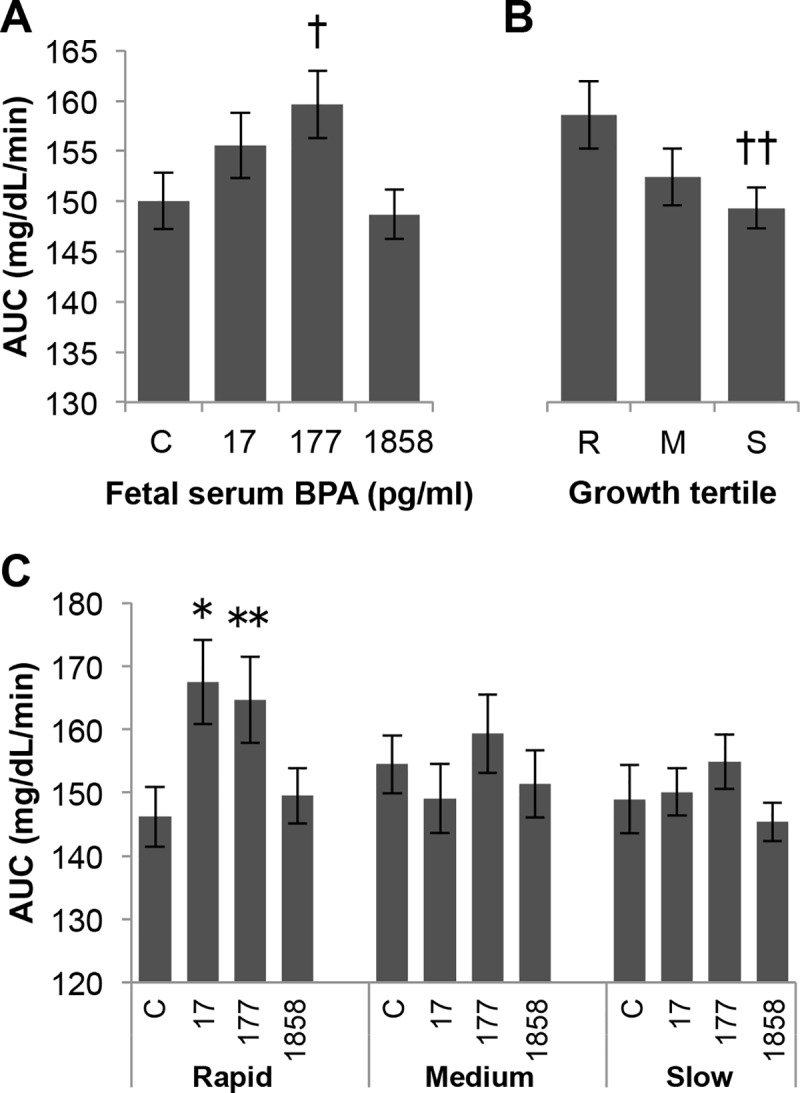
Effect of BPA and post-weaning growth on low-dose glucose tolerance test AUC. Effects on area under the blood glucose time curve (AUC) of: Panel A) treatment alone (with growth rate tertiles collapsed). Panel B) growth rate tertile alone (with treatment collapsed) and Panel C) growth rate tertile within treatment. Values are mean±SEM. ^†^P = 0.076, BPA-177 vs. controls; ^††^P = 0.087, R vs. S. * P = 0.01, rapid BPA-17 vs. rapid control; ** P = 0.055, rapid BPA-177 vs rapid control.

We also calculated the area under the blood glucose concentration time curves (AUCs) for males in the different BPA treatment groups (Fig 5A), and only the BPA-177 males had a tendency (P = 0.076) to show impaired glucose tolerance based on AUC. We also examined AUCs as a function of post-weaning growth tertile (Fig 5B), and the rapid post-weaning growth males tended to show glucose intolerance (P = 0.087) relative to the slow growth males. When the AUC data were analyzed as a function of both BPA treatment and post-weaning growth tertile (Fig 5C), we found that only the rapid growth males exposed to BPA-17 (P = 0.01) and the BPA-177 males (P = 0.055) showed evidence of impaired glucose tolerance relative to controls, while no significant effects on glucose tolerance AUCs were found for medium and slow-growth males exposed perinatally to BPA.

### Analysis of baseline blood glucose concentrations

**Fig 6:**
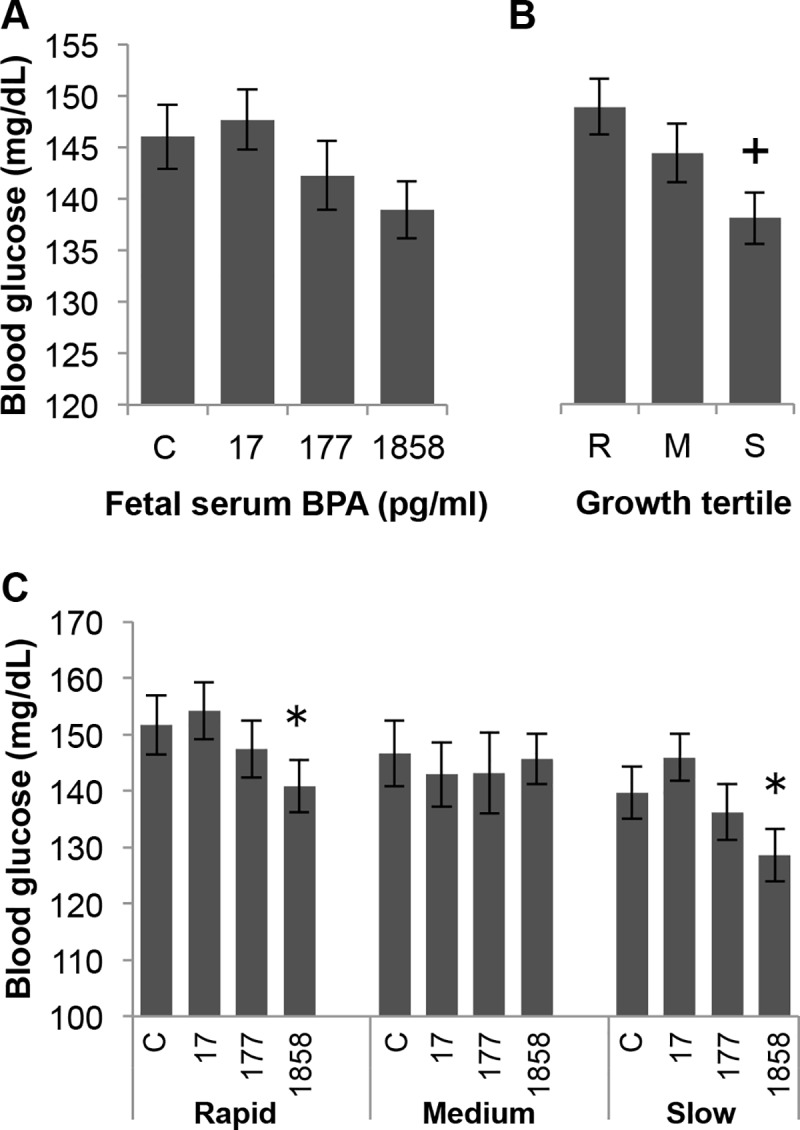
Effect of perinatal BPA exposure and post-weaning growth on baseline (Time 0) glucose concentrations. Panel A) Baseline blood glucose in controls vs. serum BPA concentrations of 17, 177 or 1858 pg/ml). Panel B) Blood glucose at baseline as a function of post-weaning growth rate tertile R (rapid), M (medium) or S (slow). Panel C) BPA treatment groups within post-weaning growth tertiles. ^+^ P < 0.01, S vs R; * P < 0.05, BPA-17 vs. BPA-1858 within both rapid and slow growth tertile groups.

The data for fasting blood glucose levels (Time 0 in the glucose tolerance test, prior to the glucose injection) are shown in Fig 6. There was a trend (P = 0.094) for an effect of BPA dose (Fig 6A), and a significant effect on fasting blood glucose levels as a function of post-weaning growth tertile (Fig 6B). Blood glucose levels were significantly lower (P < 0.01) in the slow post-weaning growth males and tended to be lower in the medium growth males (P = 0.083) compared with the rapid growth males (Fig 6B). When BPA dose and post-weaning growth were examined together, both the rapid and slow post-weaning growth males showed trends for lower fasting plasma glucose as BPA dose increased (BPA 17 vs BPA 1858, P < 0.01), while the medium growth males did not show this trend (Fig 6C).

### Percent change in blood glucose relative to baseline

**Fig 7:**
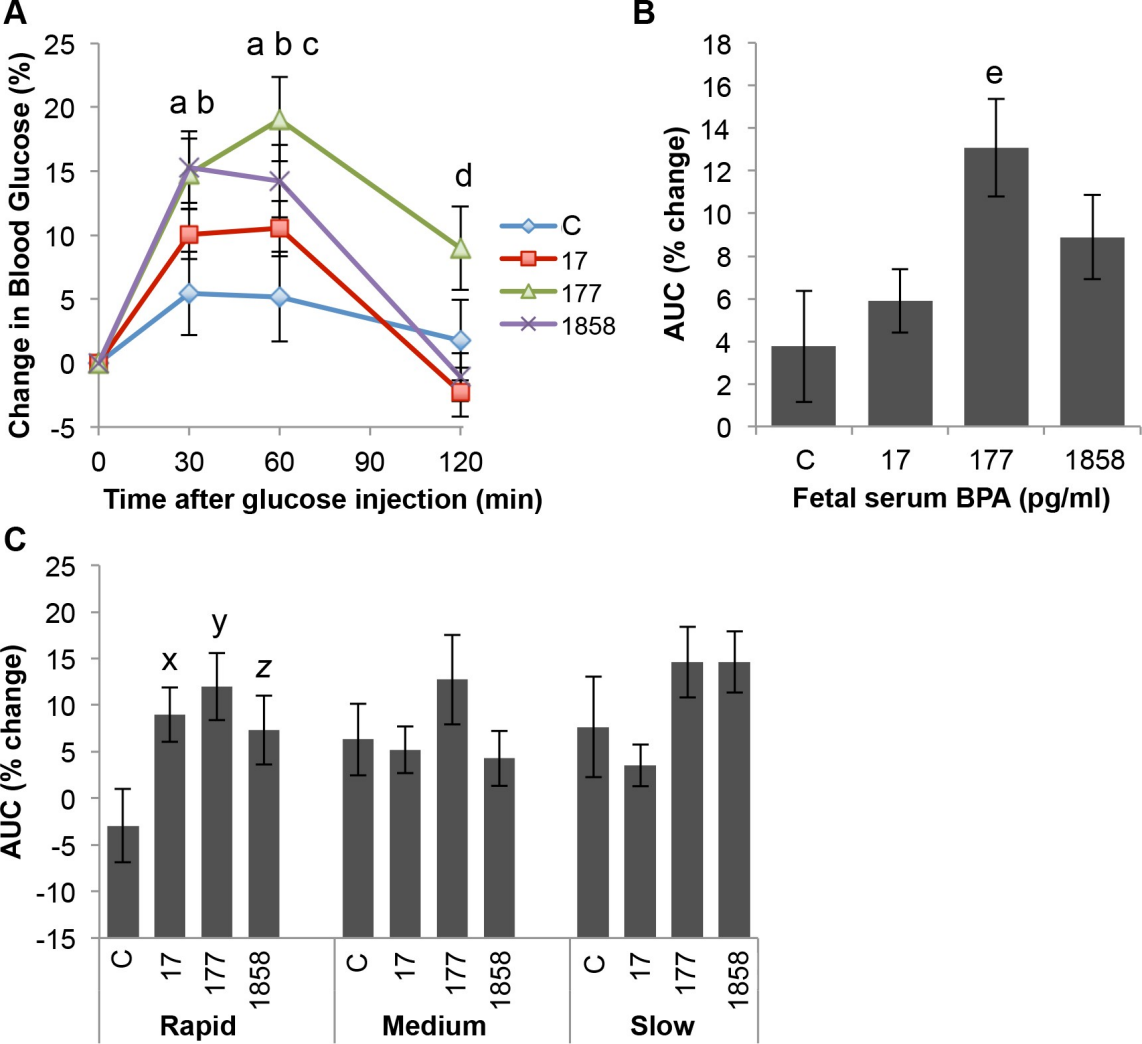
Percent change relative to baseline in blood glucose levels in response to a low-dose glucose tolerance test. Panel A) Effect of fetal serum BPA concentration alone, ignoring post-weaning growth tertile, on blood glucose levels at each timepoint after glucose injection. Panel B) Effect of fetal serum BPA concentration (ignoring growth tertile) on the area under the concentration time curve (AUC) for percent change in blood glucose levels relative to baseline in response to glucose challenge. Panel C) Effect of BPA exposure within each growth tertile group on the AUC for percent change in blood glucose relative to baseline. Values are mean±SEM. a: control vs. BPA-177 P<0.01; b: control vs. BPA-1858 P<0.05; c: control vs. BPA-17 P<0.05; d: control vs. BPA-177 P<0.05; x: rapid controls vs rapid BPA-17 P < 0.05; y: rapid controls vs. BPA-177 P < 0.05; z, rapid controls vs. rapid BPA-1858 P = 0.059.

Baseline (fasting) glucose levels varied between the four exposure groups. Thus, in order to compare the degree of response to the glucose challenge while taking baseline differences into account, we also expressed the GTT results in terms of percent change from baseline (Fig 7), using the formula [(Gx/G0)– 1)) x 100], where G0 is the glucose concentration at baseline and Gx is the glucose concentration at one of the three ensuing timepoints.

When expressed as a percent of baseline, Fig 7A shows that there was a significant treatment effect (P < 0.05). BPA-177 and BPA-1858 males had significantly higher blood glucose levels than controls at both 30 and 60 min (P < 0.01), and the BPA-17 males were also significantly different from controls at 60 min (P < 0.05). For the AUC for percent change in blood glucose (Fig 7B), ignoring post-weaning growth tertile, there was a significant treatment effect (P < 0.05), and a significant difference between controls and BPA-177 males (P < 0.01), as well as a significant (P < 0.05) difference between the BPA-177 and BPA-17 males. However, when post-weaning growth tertile was included in the analysis (Fig 7C), the rapid growth males in all of the BPA treatment groups showed evidence of effects of BPA relative to controls at all exposures on the AUC for percent change relative to baseline in plasma glucose (controls vs. BPA 17 and BPA-177, P < 0.05; controls vs. BPA-1858, P = 0.059). In contrast, medium- and slow-growth males showed no significant differences relative to controls (Fig 7C). Thus, taken together, the data in Fig 4 and Fig 7 show evidence of impaired glucose tolerance relative to controls at all BPA exposures tested in this study.

## Discussion

A major finding from our study is that in CD-1 mice, regardless of prior chemical exposure, the lightest males at weaning showed the most rapid increase in body weight over the two weeks of free feeding (between 3–5 weeks old) after weaning. There was thus a strong negative correlation between body weight at weaning and post-weaning growth rate (Fig 1). In addition, perinatal BPA exposure impacted body weight at weaning as well as the rate of post-weaning growth between weeks 3–5 and weeks 5–12 (Figs 2 & 3). The increased growth rate seen during weeks 5–12 for BPA-17 and BPA-1858 animals suggests that these males may have become obese if these elevated growth curves continued throughout life [33].

Further, body weight at weaning and rate of post-weaning growth during the first two weeks after weaning significantly predicted the effect of fetal exposure to very low doses of BPA when males were administered a glucose tolerance test (GTT) in adulthood at 12 weeks of age. Specifically, impaired glucose tolerance was seen in males exposed to the two lower doses of BPA, leading to significantly elevated serum glucose concentrations for BPA-17 and BPA-177 males (Fig 5C), and a trend of elevated glucose in BPA-1858 males (Fig 7C), but only in the males categorized as being in the rapid post-weaning growth rate tertile (with the lowest body weight at weaning). Importantly, control animals showed no response to this low dose glucose challenge. Thus, the low-dose glucose challenge revealed an increased sensitivity to glucose in all animals exposed perinatally to BPA, with the greatest effects seen in the lowest BPA exposure groups.

For males that were in the middle and slow post-weaning growth rate groups, prior BPA exposure showed little impact on the response to a glucose challenge. This finding occurred regardless of whether the BPA-treated males were compared to controls at each time point during the GTT experiment (Fig 4B) or compared based on the area under the glucose concentration-time curve (AUC) for the entire test period (Fig 5C). These findings suggest the need for prospective epidemiological studies in which effects of BPA are examined at critical periods in development, since it has already been shown that there is a relationship of growth rate during these critical growth periods and obesity and thus also type 2 diabetes in humans [6, 8], as well as a relationship between BPA levels and obesity in children [22].

In prior experimental studies, different methods for maternal administration of BPA have been used, either during prenatal life alone or during prenatal-neonatal life, complicating comparisons of dose across these studies, which is why we measured internal dose (unconjugated, bioactive BPA in serum). Our decision to use continuous exposure in this experiment is based on data indicating that human exposure to BPA is continuous and can reach levels that are likely due to non-oral sources [34–36], such as thermal receipt paper, that have not previously been taken into account by regulatory agencies [37]. Importantly, the greatest effects of fetal exposure to BPA on adult glucose tolerance occurred at exposures (Table 1) that led to fetal serum unconjugated BPA levels measured on gestation day (GD) 18 of 17.0±1.0 and 176.0±23.0 pg/ml. Because the BPA-1858 pg/ml (highest exposure) males had a somewhat lower baseline fasting glucose level relative to controls, when the AUC for the glucose concentration-time curve was calculated as a percent of baseline (Fig 7C), the BPA-1858 group also showed evidence of impaired glucose tolerance; however, as was true for the BPA-17 and BPA-177 males, this only occurred for the post-weaning rapid growth-rate males. These findings of developmental effects of BPA occurring at serum levels in the picogram per ml range clearly challenge the often-stated prediction that BPA is a “weak” endocrine disrupting chemical. The findings here are consistent with our prior findings of fetal effects of BPA on abdominal fat and numerous metabolic endpoints at similar very low pg/ml concentrations of unconjugated BPA in fetal serum following oral administration to pregnant female mice [12].

Our finding of a significant effect of developmental exposure BPA on glucose tolerance in adulthood when all males within each BPA treatment group were combined (Fig 4A; Fig 7A) is consistent with prior findings [12–16]. One difference between our study and most others is that we limited the length of the fasting time prior to conducting the GTT. In prior work (Coe, unpublished data) we noted a significant loss of body fat in mice during a 16-hr fast that we wanted to avoid in this experiment because the animals were being saved for further study. In addition, we administered a lower dose of glucose (20 mg/kg BW) than has been used in most prior studies (2 g/kg BW). A much higher glucose dose can dramatically increase blood glucose in mice [38] compared to the values obtained from the lower dose used in our study. Our low dose study may thus provide a more sensitive test of glucose tolerance. Consistent with this prediction, it is interesting to note that, in contrast to the BPA-exposed males, control males were not significantly impacted by the low glucose dose used here (Fig 4A and Fig 7A).

In another study [39], prenatal exposure of CD-1 male mice to BPA led to reduced growth and an increase in pancreatic ß cell mass in early life, followed by rapid body growth and ultimately a decrease in ß cell mass in adulthood. This study demonstrated that BPA significantly impacted proliferation and apoptosis of pancreatic cells, as well as fat and body growth trajectories. These findings may be occurring through mechanisms similar to those that led to the inverse relationship we observed between body weight at weaning and post-weaning growth rate, with males that showed the lowest body weight at weaning due to perinatal low dose BPA exposure showing the greatest impairment in glucose tolerance in adulthood. Further research will be needed to unravel the complex age-related changes in insulin secretion, insulin responsiveness and glucose homeostasis, as well as rate of growth, caused by different doses of BPA during development.

In a separate experiment we examined the relationship between body weight at birth and weaning in 293 male CD-1 mouse pups from a total of 52 litters, by individually identifying each male with a toe-clipping procedure; we showed that males that were the lightest at birth were likely (R = 0.6) to be the lightest at weaning (B. L. Coe et al., unpublished data). We did not identify individual animals at birth in the present experiment because we wanted to avoid the possibility of neonatal stress from the toe-clipping procedure, which could interact with effects of BPA exposure on subsequent metabolic parameters. However, based on our prior data, we predict that the animals in this experiment that had lowest body weight at weaning and high post-weaning weight gain, also are statistically more likely to have had the lowest body weight at birth.

Our findings here thus should be relevant to findings from epidemiological studies of light-at-term babies showing that a period of rapid “catch-up” growth in pose-weaning childhood is related to subsequent metabolic disease, including obesity, dysregulation of glucose, and an increased incidence of type 2 diabetes [6, 8]. A fetus that develops in a uterine environment in which there are reduced nutrients or placental blood flow [27] has been proposed to develop a ‘‘thrifty phenotype”, with the homeostatic mechanisms governing body weight having a “set point” that is appropriate for postnatal undernourishment but not appropriate for the typical Western high-fat diet consisting primarily of processed food. The evidence is thus that during fetal and neonatal life, the endocrine disrupting chemical BPA interacts with prenatal growth as well as postnatal growth rate to impact glucose regulation in our mouse model. Previously, fetal exposure to BPA was reported to mimic many effects of exposure to a high fat diet in male mice on metabolic endpoints [16].

The issue of the potency of endocrine disruptors has become a major point of contention in the European Union (EU) as the European Commission tries to meet the legal mandate that they develop criteria for determining whether or not a chemical should be classified as an EDC [40]. In mice, BPA has a very low potency in the uterotrophic assay [41], the assay most commonly used over the last century to assess *in vivo* estrogenic activity, and this is one basis for the controversial conclusion that BPA is a weak endocrine disrupting chemical. However, another study reported that BPA bound to estrogen receptors and stimulated an oncogene response in a vaginal proliferation assay in two strains of rats that were examined, but only one strain showed an actual increase in endothelial cell proliferation [42]. Thus, using a specific insensitive rat strain and examining an outcome that is insensitive to BPA would lead a regulatory agency to declare BPA safe for human exposure, which has been the case for the US Food and Drug Administration [43] that has relied primarily on findings using a rat strain (CD-SD) that has a low sensitivity to any estrogenic chemical [44]. In sharp contrast, we show here that examination of another critical outcome (glucose homeostasis), the disruption of which is related to a world-wide epidemic of obesity and type 2 diabetes [3], would lead to the opposite conclusion, namely, that BPA has a very high endocrine disrupting potency on metabolic systems.

In summary, we report here that an extremely low level of BPA exposure, relevant to human exposure to BPA [45] during fetal life, that led to a mean unconjugated serum concentration at the end of fetal life in mice of 17 pg/ml (0.017 ng/ml), caused a disruption of glucose homeostasis in a sensitive sub-population, males that exhibited early-life growth restriction followed by a high velocity of growth after weaning.

## Supporting information

S1 Fig The hemi-ovariectomized mouse model. (TIF)

S2 Fig Growth (body weight) from weeks 3–5 presented as growth curves. (TIFF)

S1 Table Growth velocity. (DOCX)

S2 Table Individual serum BPA concentrations. (DOCX)

S3 Table Individual body weights on weeks 3 and 5, growth rates, and blood glucose concentrations. (DOCX)
