# Supplementary figures and images for: Reduced body weight at weaning followed by increased post-weaning growth rate interacts with part-per-trillion fetal serum concentrations of bisphenol A (BPA) to impair glucose tolerance in male mice
Source: PLoS One. 2018 Dec 17;13(12):e0208846. doi: 10.1371/journal.pone.0208846 (PMC6296512; doi:10.1371/journal.pone.0208846)

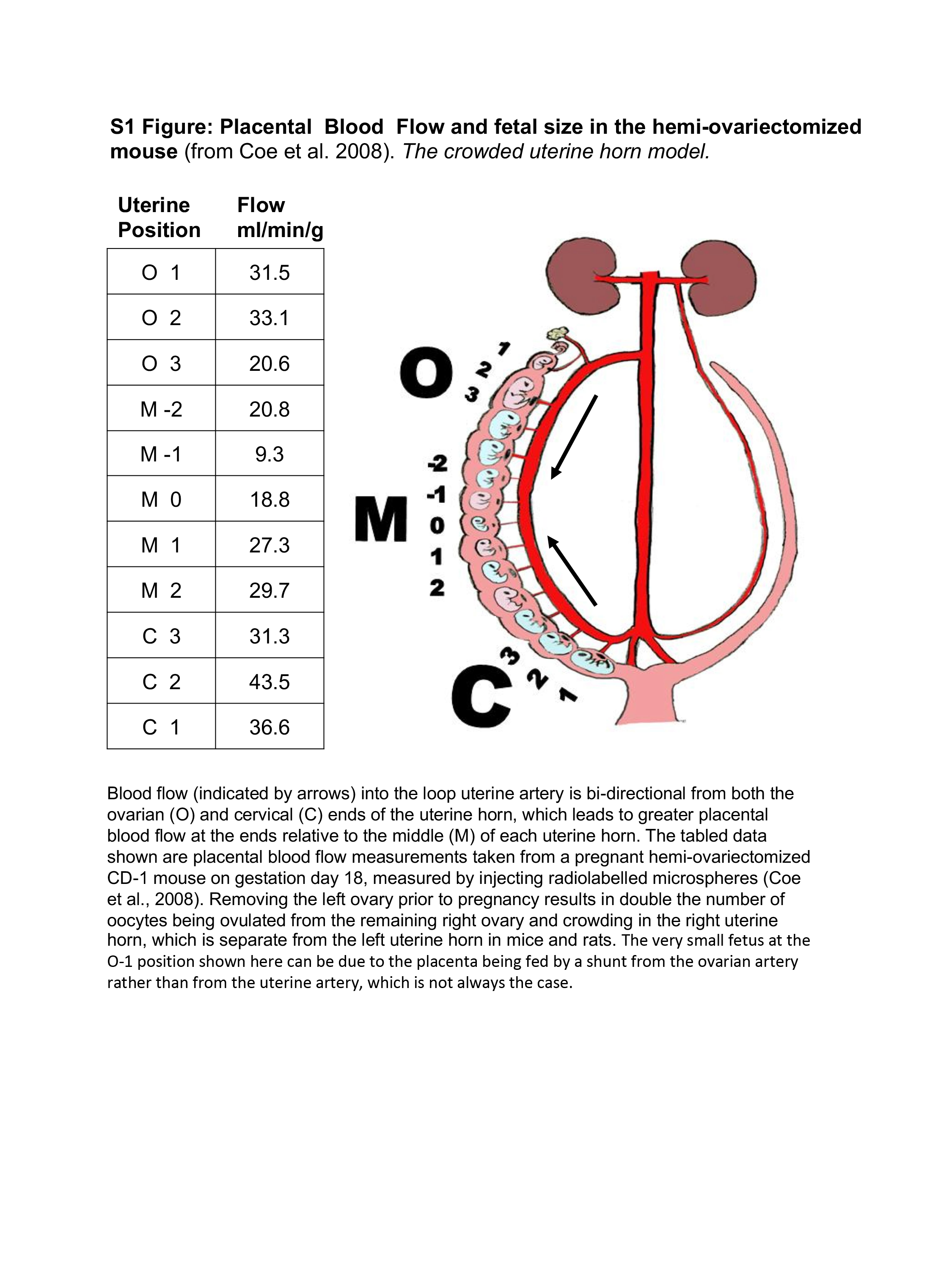

Supplement: S1 Fig — (TIF) [file pone.0208846.s001.tif]

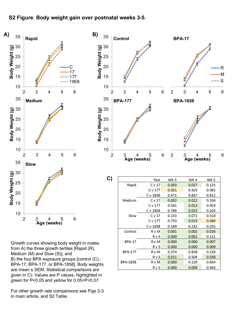

Supplement: S2 Fig — (TIFF) [file pone.0208846.s002.tiff]
