# Supplementary material for: Reduced body weight at weaning followed by increased post-weaning growth rate interacts with part-per-trillion fetal serum concentrations of bisphenol A (BPA) to impair glucose tolerance in male mice
Source: PLoS One. 2018 Dec 17;13(12):e0208846. doi: 10.1371/journal.pone.0208846 (PMC6296512; doi:10.1371/journal.pone.0208846)
Supplement: S1 Table — (DOCX) [file pone.0208846.s003.docx]

**S1 Table: Growth Velocity.**

Growth (weight gain) velocity in grams/day, by A) perinatal treatment and B) growth rate tertile. Values are mean ± SEM. Statistical tests A) compared the BPA exposure groups to control animals, either without regard to growth tertile (ALL), or individually within each tertile group, and B) compared animals in Medium and Slow growth tertile groups to animals in the Rapid category, either without regard to BPA exposure (ALL) or separately within each BPA exposure group. *P<0.05, **P<0.01, ***P<0.001. (*) 0.05<P<0.07.

|  |  |  | WEEKS 3-5 | WEEKS 5-12 |
| --- | --- | --- | --- | --- |
| A) | ALL | C | 1.24 ± 0.02 | 0.16 ± 0.01 |
|  |  | 17 | 1.25 ± 0.01 | 0.17 ± 0.01 |
|  |  | 177 | 1.17 ± 0.02 * | 0.15 ± 0.01 |
|  |  | 1858 | 1.18 ± 0.02 * | 0.19 ± 0.01 ** |
|  | Rapid | C | 1.27 ± 0.02 | 0.16 ± 0.01 |
|  |  | 17 | 1.31 ± 0.02 | 0.18 ± 0.01 |
|  |  | 177 | 1.30 ± 0.03 | 0.15 ± 0.01 |
|  |  | 1858 | 1.31 ± 0.02 * | 0.19 ± 0.01 |
|  | Medium | C | 1.28 ± 0.03 | 0.15 ± 0.01 |
|  |  | 17 | 1.27 ± 0.02 | 0.16 ± 0.01 |
|  |  | 177 | 1.17 ± 0.02 | 0.14 ± 0.01 ** |
|  |  | 1858 | 1.18 ± 0.02 (*) | 0.19 ± 0.01 * |
|  | Slow | C | 1.18 ± 0.03 | 0.17 ± 0.01 |
|  |  | 17 | 1.19 ± 0.02 | 0.17 ± 0.01 |
|  |  | 177 | 1.06 ± 0.03 | 0.14 ± 0.02 ** |
|  |  | 1858 | 1.04 ± 0.02 | 0.18 ± 0.01 *** |
| B) | ALL | Rapid | 1.3 ± 0.012 | 0.17 ± 0.01 |
|  |  | Medium | 1.23 ± 0.01 *** | 0.16 ± 0.01 |
|  |  | Slow | 1.12 ± 0.01 *** | 0.17 ± 0.01 |
|  | C | R | 1.27 ± 1.31 | 0.16 ± 0.18 |
|  |  | M | 1.28 ± 1.27 | 0.15 ± 0.16 |
|  |  | S | 1.18 ± 1.19 * | 0.17 ± 0.17 |
|  | BPA-17 | R | 1.31 ± 1.3 | 0.18 ± 0.15 |
|  |  | M | 1.27 ± 1.17 | 0.16 ± 0.14 |
|  |  | S | 1.19 ± 1.06 *** | 0.17 ± 0.14 |
|  | BPA-177 | R | 1.30 ± 1.31 | 0.15 ± 0.19 |
|  |  | M | 1.17 ± 1.18 ** | 0.14 ± 0.19 |
|  |  | S | 1.06 ± 1.04 *** | 0.14 ± 0.18 |
|  | BPA-1858 | R | 1.31 ± 0.02 | 0.19 ± 0.012 |
|  |  | M | 1.18 ± 0.03 *** | 0.19 ± 0.01 |
|  |  | S | 1.04 ± 0.03 *** | 0.18 ± 0.01 |
