# Supplementary material for: Reduced body weight at weaning followed by increased post-weaning growth rate interacts with part-per-trillion fetal serum concentrations of bisphenol A (BPA) to impair glucose tolerance in male mice
Source: PLoS One. 2018 Dec 17;13(12):e0208846. doi: 10.1371/journal.pone.0208846 (PMC6296512; doi:10.1371/journal.pone.0208846)
Supplement: S2 Table — (DOCX) [file pone.0208846.s004.docx]

**S2 Table. Individual serum BPA concentrations.**

Unconjugated and conjugated BPA concentrations in maternal and fetal/pup serum in mice perinatally exposed to BPA via maternal treatment with Silastic capsules containing 6, 60 or 600 µg 3H-BPA. Sera from pups within each litter were pooled for a single measurement per litter.

|  | BPA dose | Gestation | Maternal serum BPA (ng/ml) | | Fetal/pup serum BPA (ng/ml) | |
| --- | --- | --- | --- | --- | --- | --- |
| Litter ID | (µg/capsule) | stage | Unconjugated | Conjugated | Unconjugated | Conjugated |
| A20 | 6 µg | GD14 | 0.029 | 0.183 | . | . |
| A23 | 6 µg | GD14 | 0.016 | 0.043 | . | . |
| A31 | 6 µg | GD14 | 0.013 | 0.032 | . | . |
| A25 | 6 µg | GD18 | 0.036 | 0.029 | . | . |
| A15 | 6 µg | GD18 | 0.033 | 0.022 | 0.019 | 0.589 |
| A17 | 6 µg | GD18 | 0.024 | 0.007 | 0.016 | 0.452 |
| A18 | 6 µg | GD18 | 0.023 | 0.194 | 0.020 | 0.617 |
| A16 | 6 µg | PND2 | 0.028 | 0.103 | 0.005 | 0.051 |
| A29 | 6 µg | PND2 | 0.004 | 0.164 | 0.001 | 0.005 |
| A5 | 60 µg | GD14 | 0.244 | 0.923 | . | . |
| A6 | 60 µg | GD14 | 0.240 | 0.448 | . | . |
| A21 | 60 µg | GD14 | 0.235 | 0.974 | . | . |
| A22 | 60 µg | GD14 | 0.279 | 0.662 | . | . |
| A11 | 60 µg | GD18 | 0.202 | 0.357 | 0.139 | 3.538 |
| A12 | 60 µg | GD18 | 0.342 | 0.093 | 0.233 | 5.263 |
| A13 | 60 µg | GD18 | 0.258 | 0.547 | 0.146 | 3.592 |
| A14 | 60 µg | GD18 | 0.277 | 0.233 | 0.149 | 4.377 |
| A28 | 60 µg | PND2 | 0.287 | 2.654 | 0.114 | 0.787 |
| A30 | 60 µg | PND2 | 0.044 | 0.646 | 0.049 | 0.088 |
| A2 | 600 µg | GD14 | 4.626 | 9.661 | . | . |
| A4 | 600 µg | GD14 | 2.804 | 6.178 | . | . |
| A24 | 600 µg | GD14 | 1.314 | 8.230 | . | . |
| A56 | 600 µg | GD14 | 0.598 | 2.887 | . | . |
| A7 | 600 µg | GD18 | 2.437 | 0.065 | 1.616 | 25.334 |
| A8 | 600 µg | GD18 | 1.876 | 7.600 | 1.228 | 21.705 |
| A9 | 600 µg | GD18 | 2.329 | 2.462 | 1.746 | 25.312 |
| A10 | 600 µg | GD18 | 2.524 | 2.203 | 1.638 | 34.884 |
| A26 | 600 µg | PND2 | 1.703 | 31.926 | 0.385 | 6.995 |
| A27 | 600 µg | PND2 | 2.956 | 10.628 | 1.141 | 4.727 |
