# Supplementary material for: Reduced body weight at weaning followed by increased post-weaning growth rate interacts with part-per-trillion fetal serum concentrations of bisphenol A (BPA) to impair glucose tolerance in male mice
Source: PLoS One. 2018 Dec 17;13(12):e0208846. doi: 10.1371/journal.pone.0208846 (PMC6296512; doi:10.1371/journal.pone.0208846)
Supplement: S3 Table — (DOCX) [file pone.0208846.s005.docx]

**S3 Table. Individual body weights, growth rates and blood glucose concentrations.**

Body weights used to calculate growth rate, in male mice perinatally exposed to BPA via maternal treatment with Silastic capsules containing 0, 6, 60 or 600 µg BPA. Also shown are the blood glucose concentrations in response to a low-dose (20 mg/kg) glucose tolerance test. *AUC units are mg/dL/min.

| \| **BPA dose** \| **Animal** \| **Body weight (g**) \| \| \| **Growth** \| **Blood glucose concentration (mg/dL)** \| \| \| \| \| \| \| --- \| --- \| --- \| --- \| --- \| --- \| --- \| --- \| --- \| --- \| --- \| --- \| \| **µg/Capsule** \| ID \| Week 3 \| Week 5 \| % change \| Tertile \| 0 m \| 30 m \| 60 m \| 120 m \| AUC* \| \| 0 \| 102-2 \| 10.37 \| 29.07 \| 180.3 \| 1 \| 160 \| 130 \| 137 \| 132 \| 136.9 \| \| 0 \| 102-3 \| 11.66 \| 30.21 \| 159.1 \| 1 \| 130 \| 158 \| 96 \| 131 \| 124.3 \| \| 0 \| 120-12 \| 13.13 \| 31.42 \| 139.3 \| 1 \| 147 \| 142 \| 149 \| 132 \| 142.8 \| \| 0 \| 102-1 \| 12.56 \| 30 \| 138.9 \| 1 \| 148 \| 159 \| 169 \| 144 \| 157.6 \| \| 0 \| 102-4 \| 12.6 \| 29.93 \| 137.5 \| 1 \| 138 \| 171 \| 139 \| 146 \| 148.6 \| \| 0 \| 120-7 \| 13.37 \| 31.35 \| 134.5 \| 1 \| 182 \| 144 \| 158 \| 171 \| 160.8 \| \| 0 \| 120-13 \| 13.08 \| 30.29 \| 131.6 \| 1 \| 166 \| 138 \| 148 \| 128 \| 142.8 \| \| 0 \| 72-2 \| 13.66 \| 31.2 \| 128.4 \| 1 \| 142 \| 179 \| 171 \| 169 \| 168.9 \| \| 0 \| 120-14 \| 12.91 \| 29.48 \| 128.4 \| 1 \| 152 \| 126 \| 144 \| 115 \| 133.3 \| \| 0 \| 102-5 \| 12.92 \| 29.42 \| 127.7 \| 2 \| 145 \| 190 \| 167 \| 159 \| 168.0 \| \| 0 \| 80-5 \| 14.21 \| 32.32 \| 127.4 \| 2 \| 145 \| 166 \| 166 \| 175 \| 165.6 \| \| 0 \| 128-6 \| 15.04 \| 34.11 \| 126.8 \| 2 \| 135 \| 152 \| 150 \| 140 \| 146.1 \| \| 0 \| 80-1 \| 14.34 \| 32.41 \| 126.0 \| 2 \| 137 \| 158 \| 186 \| 185 \| 172.6 \| \| 0 \| 120-11 \| 13.49 \| 30.44 \| 125.6 \| 2 \| 160 \| 136 \| 137 \| 133 \| 138.6 \| \| 0 \| 72-3 \| 15.11 \| 34.03 \| 125.2 \| 2 \| 192 \| 178 \| 184 \| 149 \| 174.8 \| \| 0 \| 120-8 \| 13.29 \| 29.76 \| 123.9 \| 2 \| 136 \| 143 \| 129 \| 137 \| 135.4 \| \| 0 \| 128-7 \| 16.10 \| 35.96 \| 123.4 \| 2 \| 125 \| 150 \| 148 \| 146 \| 145.1 \| \| 0 \| 128-10 \| 14.97 \| 33.06 \| 120.8 \| 2 \| 150 \| 161 \| 157 \| 139 \| 152.6 \| \| 0 \| 80-3 \| 14.02 \| 30.49 \| 117.5 \| 2 \| 141 \| 140 \| 156 \| 140 \| 146.1 \| \| 0 \| 72-1 \| 14.9 \| 32.19 \| 116.0 \| 3 \| 121 \| 153 \| 178 \| 152 \| 158.1 \| \| 0 \| 120-9 \| 14.23 \| 30.31 \| 113.0 \| 3 \| 151 \| 172 \| 165 \| 166 \| 165.3 \| \| 0 \| 128-5 \| 16.21 \| 34.33 \| 111.8 \| 3 \| 151 \| 125 \| 112 \| 163 \| 132.9 \| \| 0 \| 80-2 \| 13.38 \| 28.28 \| 111.4 \| 3 \| 134 \| 128 \| 151 \| 181 \| 150.6 \| \| 0 \| 72-4 \| 15.41 \| 32.51 \| 111.0 \| 3 \| 135 \| 188 \| 196 \| 155 \| 176.1 \| \| 0 \| 128-9 \| 15.54 \| 32.75 \| 110.7 \| 3 \| 156 \| 139 \| 147 \| 145 \| 145.6 \| \| 0 \| 80-4 \| 13.48 \| 28.34 \| 110.2 \| 3 \| 140 \| 164 \| 153 \| 145 \| 152.1 \| \| 0 \| 120-10 \| 15.73 \| 31.89 \| 102.7 \| 3 \| 151 \| 135 \| 132 \| 117 \| 131.4 \| \| 0 \| 128-8 \| 17.26 \| 33.93 \| 96.6 \| 3 \| 118 \| 135 \| 133 \| 120 \| 128.4 \| \| 6 \| 85-7 \| 6.83 \| 25.65 \| 275.5 \| 1 \| 159 \| 173 \| 157 \| 137 \| 156.3 \| \| 6 \| 89-1 \| 8.22 \| 25.83 \| 214.2 \| 1 \| 124 \| 152 \| 147 \| 115 \| 137.4 \| \| 6 \| 85-10 \| 7.82 \| 24.43 \| 212.4 \| 1 \| 176 \| 208 \| 216 \| 170 \| 197.5 \| \| 6 \| 85-2 \| 9.43 \| 29.13 \| 208.9 \| 1 \| 154 \| 194 \| 222 \| 172 \| 194.0 \| \| 6 \| 85-1 \| 9.97 \| 29.84 \| 199.3 \| 1 \| 159 \| 213 \| 207 \| 211 \| 203.5 \| \| 6 \| 85-6 \| 10.91 \| 31.4 \| 187.8 \| 1 \| 182 \| 215 \| 230 \| 215 \| 216.5 \| \| 6 \| 85-4 \| 10.68 \| 30.29 \| 183.6 \| 1 \| 146 \| 176 \| 190 \| 169 \| 175.8 \| \| 6 \| 138-9 \| 8.90 \| 25.17 \| 182.8 \| 1 \| 121 \| 155 \| 153 \| 132 \| 144.3 \| \| 6 \| 85-8 \| 11.35 \| 31.8 \| 180.2 \| 1 \| 163 \| 165 \| 177 \| 126 \| 159.5 \| \| 6 \| 85-5 \| 10.74 \| 29.68 \| 176.4 \| 1 \| 206 \| 311 \| 251 \| 163 \| 238.4 \| \| 6 \| 84-7 \| 8.6 \| 23.67 \| 175.2 \| 1 \| 132 \| 134 \| 140 \| 140 \| 137.5 \| \| 6 \| 85-9 \| 11.6 \| 31.5 \| 171.6 \| 1 \| 153 \| 145 \| 195 \| 169 \| 170.8 \| \| 6 \| 85-3 \| 11.4 \| 30.87 \| 170.8 \| 1 \| 147 \| 169 \| 190 \| 183 \| 177.6 \| \| 6 \| 138-11 \| 11.27 \| 28.91 \| 156.5 \| 1 \| 201 \| 211 \| 193 \| 182 \| 195.8 \| \| 6 \| 140-6 \| 11.61 \| 29.67 \| 155.6 \| 1 \| 160 \| 145 \| 144 \| 144 \| 146.3 \| \| 6 \| 140-8 \| 11.88 \| 30.05 \| 152.9 \| 1 \| 161 \| 141 \| 97 \| 82 \| 112.3 \| \| 6 \| 76-2 \| 12.43 \| 31.12 \| 150.4 \| 1 \| 118 \| 176 \| 135 \| 138 \| 143.9 \| \| 6 \| 84-2 \| 12.22 \| 30.35 \| 148.4 \| 1 \| 148 \| 161 \| 209 \| 139 \| 171.9 \| \| 6 \| 143-5 \| 11.29 \| 28.01 \| 148.1 \| 1 \| 118 \| 122 \| 121 \| 122 \| 121.1 \| \| 6 \| 138-7 \| 11.59 \| 28.25 \| 143.7 \| 1 \| 160 \| 148 \| 187 \| 161 \| 167.4 \| \| 6 \| 140-4 \| 13.11 \| 31.93 \| 143.6 \| 1 \| 161 \| 182 \| 135 \| 135 \| 150.0 \| \| 6 \| 84-6 \| 12.36 \| 29.89 \| 141.8 \| 1 \| 143 \| 169 \| 200 \| 126 \| 166.6 \| \| 6 \| 138-14 \| 12.18 \| 29.42 \| 141.5 \| 2 \| 186 \| 203 \| 161 \| 164 \| 175.4 \| \| 6 \| 81-2 \| 13.91 \| 33.51 \| 140.9 \| 2 \| 139 \| 149 \| 161 \| 132 \| 148.0 \| \| 6 \| 76-1 \| 13.07 \| 31.46 \| 140.7 \| 2 \| 162 \| 180 \| 196 \| 195 \| 187.5 \| \| 6 \| 138-12 \| 12.56 \| 30.11 \| 139.7 \| 2 \| 190 \| 198 \| 173 \| 157 \| 177.4 \| \| 6 \| 140-5 \| 13.04 \| 31.13 \| 138.7 \| 2 \| 148 \| 196 \| 150 \| 123 \| 154.5 \| \| 6 \| 76-4 \| 12.62 \| 30.12 \| 138.7 \| 2 \| 154 \| 157 \| 160 \| 184 \| 164.5 \| \| 6 \| 140-7 \| 13.14 \| 31.36 \| 138.7 \| 2 \| 175 \| 167 \| 177 \| 158 \| 169.5 \| \| 6 \| 143-4 \| 12.27 \| 29.25 \| 138.4 \| 2 \| 133 \| 135 \| 140 \| 128 \| 134.9 \| \| 6 \| 143-9 \| 13.19 \| 31.41 \| 138.1 \| 2 \| 136 \| 134 \| 146 \| 125 \| 136.5 \| \| 6 \| 143-12 \| 12.87 \| 30.38 \| 136.1 \| 2 \| 122 \| 121 \| 114 \| 97 \| 112.5 \| \| 6 \| 84-1 \| 13.17 \| 31.04 \| 135.7 \| 2 \| 147 \| 136 \| 151 \| 122 \| 139.5 \| \| 6 \| 143-8 \| 12.72 \| 29.91 \| 135.1 \| 2 \| 94 \| 117 \| 134 \| 98 \| 115.8 \| \| 6 \| 76-5 \| 13.48 \| 31.55 \| 134.1 \| 2 \| 140 \| 202 \| 176 \| 204 \| 185.0 \| \| 6 \| 140-12 \| 13.45 \| 31.26 \| 132.4 \| 2 \| 156 \| 141 \| 145 \| 159 \| 148.9 \| \| 6 \| 81-4 \| 12.32 \| 28.5 \| 131.3 \| 2 \| 163 \| 187 \| 189 \| 168 \| 180.0 \| \| 6 \| 143-10 \| 12.62 \| 29.07 \| 130.3 \| 2 \| 85 \| 94 \| 88 \| 82 \| 87.6 \| \| 6 \| 140-9 \| 14.59 \| 33.55 \| 130.0 \| 2 \| 166 \| 142 \| 149 \| 141 \| 147.4 \| \| 6 \| 76-3 \| 13.24 \| 30.43 \| 129.8 \| 2 \| 155 \| 146 \| 159 \| 142 \| 151.0 \| \| 6 \| 84-4 \| 12.88 \| 29.56 \| 129.5 \| 2 \| 126 \| 172 \| 154 \| 147 \| 153.3 \| \| 6 \| 86-1 \| 16.42 \| 37.5 \| 128.4 \| 2 \| 134 \| 149 \| 143 \| 147 \| 144.4 \| \| 6 \| 138-8 \| 13.41 \| 30.49 \| 127.4 \| 2 \| 120 \| 152 \| 155 \| 150 \| 148.6 \| \| 6 \| 143-11 \| 12.70 \| 28.81 \| 126.9 \| 2 \| 113 \| 131 \| 113 \| 113 \| 117.5 \| \| 6 \| 138-13 \| 11.77 \| 26.59 \| 125.9 \| 3 \| 161 \| 186 \| 184 \| 172 \| 178.6 \| \| 6 \| 81-1 \| 15.07 \| 33.95 \| 125.3 \| 3 \| 141 \| 155 \| 177 \| 145 \| 159.0 \| \| 6 \| 140-11 \| 13.60 \| 30.63 \| 125.2 \| 3 \| 171 \| 143 \| 157 \| 150 \| 153.5 \| \| 6 \| 140-10 \| 13.03 \| 29.15 \| 123.7 \| 3 \| 158 \| 175 \| 153 \| 139 \| 155.6 \| \| 6 \| 89-3 \| 14.16 \| 31.6 \| 123.2 \| 3 \| 139 \| 179 \| 139 \| 120 \| 144.3 \| \| 6 \| 84-9 \| 13.19 \| 29.35 \| 122.5 \| 3 \| 129 \| 153 \| 203 \| 125 \| 161.6 \| \| 6 \| 143-14 \| 12.60 \| 27.58 \| 118.9 \| 3 \| 127 \| 126 \| 126 \| 140 \| 129.6 \| \| 6 \| 138-10 \| 12.95 \| 28.23 \| 118.0 \| 3 \| 153 \| 164 \| 193 \| 136 \| 166.5 \| \| 6 \| 84-8 \| 14.88 \| 32.43 \| 117.9 \| 3 \| 137 \| 153 \| 185 \| 132 \| 157.8 \| \| 6 \| 86-7 \| 15 \| 32.5 \| 116.7 \| 3 \| 125 \| 140 \| 147 \| 135 \| 139.5 \| \| 6 \| 143-6 \| 12.96 \| 27.98 \| 115.9 \| 3 \| 118 \| 128 \| 143 \| 134 \| 133.9 \| \| 6 \| 81-3 \| 14.41 \| 31.03 \| 115.3 \| 3 \| 158 \| 202 \| 174 \| 128 \| 167.5 \| \| 6 \| 86-3 \| 15.9 \| 34.21 \| 115.2 \| 3 \| 190 \| 162 \| 174 \| 150 \| 167.0 \| \| 6 \| 86-4 \| 15.52 \| 33.38 \| 115.1 \| 3 \| 139 \| 140 \| 150 \| 140 \| 143.6 \| \| 6 \| 84-3 \| 14.1 \| 30.29 \| 114.8 \| 3 \| 123 \| 151 \| 126 \| 124 \| 131.4 \| \| 6 \| 86-5 \| 15.68 \| 33.41 \| 113.1 \| 3 \| 131 \| 134 \| 130 \| 117 \| 127.9 \| \| 6 \| 89-4 \| 14.73 \| 31.32 \| 112.6 \| 3 \| 174 \| 161 \| 155 \| 134 \| 153.6 \| \| 6 \| 89-2 \| 15.29 \| 32.47 \| 112.4 \| 3 \| 155 \| 198 \| 173 \| 156 \| 172.8 \| \| 6 \| 86-6 \| 16.28 \| 34.44 \| 111.5 \| 3 \| 153 \| 145 \| 149 \| 130 \| 143.8 \| \| 6 \| 84-5 \| 15.01 \| 31.25 \| 108.2 \| 3 \| 147 \| 176 \| 179 \| 133 \| 162.8 \| \| 6 \| 143-7 \| 13.24 \| 27.51 \| 107.8 \| 3 \| 118 \| 99 \| 100 \| 101 \| 102.3 \| \| 6 \| 86-2 \| 16.25 \| 32.66 \| 101.0 \| 3 \| 164 \| 141 \| 159 \| 142 \| 150.9 \| \| 60 \| 90-4 \| 12.3 \| 30.61 \| 148.9 \| 1 \| 138 \| 181 \| 156 \| 116 \| 150.0 \| \| 60 \| 71-1 \| 11.78 \| 29 \| 146.2 \| 1 \| 177 \| 206 \| 221 \| 200 \| 206.5 \| \| 60 \| 90-3 \| 13.29 \| 30.93 \| 132.7 \| 1 \| 161 \| 158 \| 172 \| 166 \| 165.6 \| \| 60 \| 90-7 \| 13.76 \| 31.97 \| 132.3 \| 1 \| 125 \| 139 \| 137 \| 120 \| 131.8 \| \| 60 \| 71-3 \| 15.03 \| 34.88 \| 132.1 \| 1 \| 157 \| 198 \| 193 \| 154 \| 180.0 \| \| 60 \| 78-2 \| 13.69 \| 31.42 \| 129.5 \| 1 \| 131 \| 174 \| 168 \| 137 \| 157.1 \| \| 60 \| 78-5 \| 13.96 \| 31.96 \| 128.9 \| 1 \| 148 \| 165 \| 163 \| 164 \| 161.9 \| \| 60 \| 78-4 \| 13.77 \| 31.45 \| 128.4 \| 1 \| 135 \| 167 \| 204 \| 209 \| 187.4 \| \| 60 \| 73-1 \| 16.02 \| 36.52 \| 128.0 \| 1 \| 144 \| 158 \| 122 \| 170 \| 145.8 \| \| 60 \| 78-1 \| 13.18 \| 29.55 \| 124.2 \| 1 \| 158 \| 164 \| 158 \| 164 \| 161.0 \| \| 60 \| 71-2 \| 13.06 \| 29.16 \| 123.3 \| 2 \| 136 \| 209 \| 167 \| 190 \| 179.4 \| \| 60 \| 90-5 \| 13.46 \| 30.01 \| 123.0 \| 2 \| 125 \| 143 \| 140 \| 119 \| 133.6 \| \| 60 \| 78-3 \| 13.44 \| 29.59 \| 120.2 \| 2 \| 103 \| 140 \| 163 \| 125 \| 140.3 \| \| 60 \| 90-2 \| 13.7 \| 30.11 \| 119.8 \| 2 \| 149 \| 188 \| 197 \| 211 \| 192.3 \| \| 60 \| 125-13 \| 15.33 \| 33.36 \| 117.6 \| 2 \| 136 \| 135 \| 141 \| 123 \| 134.4 \| \| 60 \| 90-6 \| 14.51 \| 31.41 \| 116.5 \| 2 \| 152 \| 164 \| 176 \| 141 \| 161.3 \| \| 60 \| 71-4 \| 14.85 \| 31.67 \| 113.3 \| 2 \| 170 \| 160 \| 166 \| 153 \| 161.8 \| \| 60 \| 125-12 \| 13.88 \| 29.52 \| 112.7 \| 2 \| 150 \| 164 \| 163 \| 155 \| 159.6 \| \| 60 \| 107-2 \| 13.55 \| 28.26 \| 108.6 \| 2 \| 181 \| 176 \| 188 \| 158 \| 176.7 \| \| 60 \| 125-11 \| 15.21 \| 31.64 \| 108.0 \| 2 \| 129 \| 139 \| 163 \| 169 \| 154.3 \| \| 60 \| 107-3 \| 13.79 \| 28.54 \| 107.0 \| 3 \| 140 \| 146 \| 144 \| 129 \| 140.3 \| \| 60 \| 78-6 \| 14.44 \| 29.75 \| 106.0 \| 3 \| 138 \| 140 \| 155 \| 134 \| 143.9 \| \| 60 \| 73-3 \| 16.3 \| 33.26 \| 104.0 \| 3 \| 165 \| 130 \| 163 \| 184 \| 160.3 \| \| 60 \| 73-2 \| 15.78 \| 32.07 \| 103.2 \| 3 \| 132 \| 171 \| 181 \| 171 \| 169.9 \| \| 60 \| 107-4 \| 13.86 \| 28.09 \| 102.7 \| 3 \| 134 \| 158 \| 220 \| 141 \| 174.0 \| \| 60 \| 107-1 \| 14.98 \| 29.84 \| 99.2 \| 3 \| 146 \| 165 \| 172 \| 152 \| 162.0 \| \| 60 \| 125-10 \| 15.32 \| 30.41 \| 98.5 \| 3 \| 122 \| 143 \| 153 \| 143 \| 144.1 \| \| 60 \| 125-9 \| 15.29 \| 29.71 \| 94.3 \| 3 \| 114 \| 150 \| 155 \| 151 \| 147.6 \| \| 60 \| 107-5 \| 15.05 \| 28.65 \| 90.4 \| 3 \| 152 \| 182 \| 172 \| 162 \| 169.5 \| \| 60 \| 125-8 \| 14.92 \| 27.18 \| 82.2 \| 3 \| 119 \| 144 \| 157 \| 112 \| 137.8 \| \| 600 \| 88-10 \| 7.79 \| 25.48 \| 227.1 \| 1 \| . \| . \| . \| . \| . \| \| 600 \| 88-6 \| 11.3 \| 31.74 \| 180.9 \| 1 \| 165 \| 182 \| 168 \| 145 \| 165.4 \| \| 600 \| 88-1 \| 10.76 \| 29.94 \| 178.3 \| 1 \| 152 \| 148 \| 131 \| 104 \| 131.1 \| \| 600 \| 88-9 \| 10.58 \| 29.02 \| 174.3 \| 1 \| . \| . \| . \| . \| . \| \| 600 \| 88-8 \| 11.33 \| 30.08 \| 165.5 \| 1 \| 154 \| 202 \| 203 \| 155 \| 184.6 \| \| 600 \| 88-7 \| 12.13 \| 31.87 \| 162.7 \| 1 \| 150 \| 193 \| 150 \| 115 \| 152.0 \| \| 600 \| 88-2 \| 12.22 \| 31.94 \| 161.4 \| 1 \| 166 \| 163 \| 159 \| 121 \| 151.4 \| \| 600 \| 88-5 \| 11.9 \| 30.66 \| 157.6 \| 1 \| 154 \| 188 \| 170 \| 151 \| 167.8 \| \| 600 \| 74-4 \| 11.56 \| 29.61 \| 156.1 \| 1 \| 138 \| 136 \| 144 \| 125 \| 136.5 \| \| 600 \| 103-6 \| 10.08 \| 24.95 \| 147.5 \| 1 \| 134 \| 144 \| 145 \| 125 \| 138.4 \| \| 600 \| 74-2 \| 12.9 \| 30.92 \| 139.7 \| 1 \| 117 \| 178 \| 158 \| 134 \| 151.9 \| \| 600 \| 103-5 \| 12.72 \| 30.21 \| 137.5 \| 1 \| 160 \| 159 \| 142 \| 134 \| 146.5 \| \| 600 \| 74-1 \| 13.84 \| 32.59 \| 135.5 \| 1 \| 108 \| 163 \| 164 \| 134 \| 149.3 \| \| 600 \| 74-5 \| 12.74 \| 29.81 \| 134.0 \| 1 \| 118 \| 123 \| 114 \| 133 \| 121.5 \| \| 600 \| 74-3 \| 13.87 \| 32.42 \| 133.7 \| 1 \| 125 \| 122 \| 132 \| 117 \| 124.9 \| \| 600 \| 123-10 \| 13.57 \| 31.61 \| 132.9 \| 1 \| 143 \| 168 \| 191 \| 180 \| 176.5 \| \| 600 \| 92-6 \| 13.82 \| 31.68 \| 129.2 \| 1 \| 151 \| 158 \| 154 \| 140 \| 151.1 \| \| 600 \| 92-2 \| 15.28 \| 34.94 \| 128.7 \| 1 \| 119 \| 150 \| 159 \| 127 \| 143.8 \| \| 600 \| 127-10 \| 12.46 \| 28.39 \| 127.8 \| 2 \| 129 \| 130 \| 193 \| 132 \| 154.0 \| \| 600 \| 127-11 \| 12.52 \| 28.50 \| 127.6 \| 2 \| 138 \| 150 \| 216 \| 141 \| 171.0 \| \| 600 \| 92-3 \| 14.41 \| 32.6 \| 126.2 \| 2 \| 144 \| 154 \| 160 \| 162 \| 157.0 \| \| 600 \| 103-3 \| 13.6 \| 30.45 \| 123.9 \| 2 \| 154 \| 147 \| 137 \| 128 \| 139.4 \| \| 600 \| 103-8 \| 15.14 \| 33.59 \| 121.9 \| 2 \| 164 \| 165 \| 137 \| 150 \| 150.6 \| \| 600 \| 103-7 \| 14.65 \| 32.06 \| 118.8 \| 2 \| 146 \| 132 \| 126 \| 122 \| 129.0 \| \| 600 \| 123-8 \| 12.68 \| 27.64 \| 118.0 \| 2 \| 111 \| 147 \| 157 \| 110 \| 137.0 \| \| 600 \| 92-5 \| 14.83 \| 32.19 \| 117.1 \| 2 \| 142 \| 168 \| 144 \| 133 \| 147.0 \| \| 600 \| 92-1 \| 15.57 \| 33.46 \| 114.9 \| 2 \| 163 \| 157 \| 172 \| 137 \| 158.4 \| \| 600 \| 103-4 \| 15.41 \| 32.8 \| 112.8 \| 2 \| 162 \| 171 \| 172 \| 162 \| 168.0 \| \| 600 \| 123-11 \| 14.82 \| 31.52 \| 112.7 \| 2 \| 156 \| 192 \| 188 \| 171 \| 180.8 \| \| 600 \| 123-12 \| 14.32 \| 30.43 \| 112.5 \| 2 \| 141 \| 172 \| 166 \| 138 \| 157.4 \| \| 600 \| 92-4 \| 14.37 \| 30.05 \| 109.1 \| 2 \| 134 \| 142 \| 151 \| 133 \| 142.1 \| \| 600 \| 127-12 \| 13.77 \| 28.63 \| 107.9 \| 2 \| 155 \| 141 \| 143 \| 137 \| 142.5 \| \| 600 \| 103-2 \| 14.55 \| 30.23 \| 107.8 \| 2 \| 140 \| 152 \| 154 \| 142 \| 148.8 \| \| 600 \| 106-6 \| 12.51 \| 25.91 \| 107.1 \| 2 \| 113 \| 109 \| 80 \| 99 \| 95.9 \| \| 600 \| 106-1 \| 16.12 \| 33.32 \| 106.7 \| 2 \| 185 \| 209 \| 208 \| 167 \| 195.1 \| \| 600 \| 137-10 \| 13.05 \| 26.54 \| 103.4 \| 3 \| 137 \| 142 \| 135 \| 137 \| 137.5 \| \| 600 \| 137-4 \| 15.40 \| 30.78 \| 99.9 \| 3 \| 114 \| 167 \| 134 \| 115 \| 135.0 \| \| 600 \| 103-1 \| 14.19 \| 28.14 \| 98.3 \| 3 \| 160 \| 168 \| 158 \| 159 \| 161.0 \| \| 600 \| 130-6 \| 17.33 \| 34.35 \| 98.2 \| 3 \| 110 \| 146 \| 169 \| 114 \| 142.1 \| \| 600 \| 106-2 \| 15.74 \| 31.12 \| 97.7 \| 3 \| 147 \| 184 \| 183 \| 154 \| 171.5 \| \| 600 \| 106-3 \| 15.05 \| 29.54 \| 96.3 \| 3 \| 160 \| 143 \| 165 \| 131 \| 150.4 \| \| 600 \| 137-5 \| 15.72 \| 30.78 \| 95.8 \| 3 \| 104 \| 206 \| 172 \| 129 \| 161.3 \| \| 600 \| 137-6 \| 15.18 \| 29.51 \| 94.4 \| 3 \| 125 \| 184 \| 137 \| 139 \| 147.8 \| \| 600 \| 137-7 \| 16.29 \| 31.44 \| 93.0 \| 3 \| 113 \| 141 \| 145 \| 119 \| 133.5 \| \| 600 \| 123-9 \| 15.04 \| 28.70 \| 90.8 \| 3 \| 132 \| 136 \| 154 \| 126 \| 139.8 \| \| 600 \| 137-8 \| 15.80 \| 30.11 \| 90.6 \| 3 \| 140 \| 149 \| 155 \| 147 \| 149.6 \| \| 600 \| 106-5 \| 16.36 \| 30.57 \| 86.9 \| 3 \| 165 \| 178 \| 170 \| 141 \| 164.1 \| \| 600 \| 130-4 \| 17.59 \| 32.67 \| 85.7 \| 3 \| 123 \| 137 \| 143 \| 128 \| 135.3 \| \| 600 \| 130-9 \| 19.19 \| 35.54 \| 85.2 \| 3 \| 123 \| 142 \| 142 \| 140 \| 139.1 \| \| 600 \| 130-8 \| 17.76 \| 32.35 \| 82.2 \| 3 \| 126 \| 135 \| 168 \| 108 \| 139.5 \| \| 600 \| 137-9 \| 14.56 \| 25.91 \| 78.0 \| 3 \| 117 \| 156 \| 142 \| 146 \| 143.4 \| \| 600 \| 106-4 \| 17.12 \| 29.89 \| 74.6 \| 3 \| 120 \| 158 \| 159 \| 136 \| 148.1 \| \| 600 \| 130-5 \| 17.77 \| 30.83 \| 73.5 \| 3 \| 98 \| 127 \| 122 \| 114 \| 118.3 \| |
| --- | --- | --- | --- | --- | --- | --- | --- | --- | --- | --- | --- | --- | --- | --- | --- | --- | --- | --- | --- | --- | --- | --- | --- | --- | --- | --- | --- | --- | --- | --- | --- | --- | --- | --- | --- | --- | --- | --- | --- | --- | --- | --- | --- | --- | --- | --- | --- | --- | --- | --- | --- | --- | --- | --- | --- | --- | --- | --- | --- | --- | --- | --- | --- | --- | --- | --- | --- | --- | --- | --- | --- | --- | --- | --- | --- | --- | --- | --- | --- | --- | --- | --- | --- | --- | --- | --- | --- | --- | --- | --- | --- | --- | --- | --- | --- | --- | --- | --- | --- | --- | --- | --- | --- | --- | --- | --- | --- | --- | --- | --- | --- | --- | --- | --- | --- | --- | --- | --- | --- | --- | --- | --- | --- | --- | --- | --- | --- | --- | --- | --- | --- | --- | --- | --- | --- | --- | --- | --- | --- | --- | --- | --- | --- | --- | --- | --- | --- | --- | --- | --- | --- | --- | --- | --- | --- | --- | --- | --- | --- | --- | --- | --- | --- | --- | --- | --- | --- | --- | --- | --- | --- | --- | --- | --- | --- | --- | --- | --- | --- | --- | --- | --- | --- | --- | --- | --- | --- | --- | --- | --- | --- | --- | --- | --- | --- | --- | --- | --- | --- | --- | --- | --- | --- | --- | --- | --- | --- | --- | --- | --- | --- | --- | --- | --- | --- | --- | --- | --- | --- | --- | --- | --- | --- | --- | --- | --- | --- | --- | --- | --- | --- | --- | --- | --- | --- | --- | --- | --- | --- | --- | --- | --- | --- | --- | --- | --- | --- | --- | --- | --- | --- | --- | --- | --- | --- | --- | --- | --- | --- | --- | --- | --- | --- | --- | --- | --- | --- | --- | --- | --- | --- | --- | --- | --- | --- | --- | --- | --- | --- | --- | --- | --- | --- | --- | --- | --- | --- | --- | --- | --- | --- | --- | --- | --- | --- | --- | --- | --- | --- | --- | --- | --- | --- | --- | --- | --- | --- | --- | --- | --- | --- | --- | --- | --- | --- | --- | --- | --- | --- | --- | --- | --- | --- | --- | --- | --- | --- | --- | --- | --- | --- | --- | --- | --- | --- | --- | --- | --- | --- | --- | --- | --- | --- | --- | --- | --- | --- | --- | --- | --- | --- | --- | --- | --- | --- | --- | --- | --- | --- | --- | --- | --- | --- | --- | --- | --- | --- | --- | --- | --- | --- | --- | --- | --- | --- | --- | --- | --- | --- | --- | --- | --- | --- | --- | --- | --- | --- | --- | --- | --- | --- | --- | --- | --- | --- | --- | --- | --- | --- | --- | --- | --- | --- | --- | --- | --- | --- | --- | --- | --- | --- | --- | --- | --- | --- | --- | --- | --- | --- | --- | --- | --- | --- | --- | --- | --- | --- | --- | --- | --- | --- | --- | --- | --- | --- | --- | --- | --- | --- | --- | --- | --- | --- | --- | --- | --- | --- | --- | --- | --- | --- | --- | --- | --- | --- | --- | --- | --- | --- | --- | --- | --- | --- | --- | --- | --- | --- | --- | --- | --- | --- | --- | --- | --- | --- | --- | --- | --- | --- | --- | --- | --- | --- | --- | --- | --- | --- | --- | --- | --- | --- | --- | --- | --- | --- | --- | --- | --- | --- | --- | --- | --- | --- | --- | --- | --- | --- | --- | --- | --- | --- | --- | --- | --- | --- | --- | --- | --- | --- | --- | --- | --- | --- | --- | --- | --- | --- | --- | --- | --- | --- | --- | --- | --- | --- | --- | --- | --- | --- | --- | --- | --- | --- | --- | --- | --- | --- | --- | --- | --- | --- | --- | --- | --- | --- | --- | --- | --- | --- | --- | --- | --- | --- | --- | --- | --- | --- | --- | --- | --- | --- | --- | --- | --- | --- | --- | --- | --- | --- | --- | --- | --- | --- | --- | --- | --- | --- | --- | --- | --- | --- | --- | --- | --- | --- | --- | --- | --- | --- | --- | --- | --- | --- | --- | --- | --- | --- | --- | --- | --- | --- | --- | --- | --- | --- | --- | --- | --- | --- | --- | --- | --- | --- | --- | --- | --- | --- | --- | --- | --- | --- | --- | --- | --- | --- | --- | --- | --- | --- | --- | --- | --- | --- | --- | --- | --- | --- | --- | --- | --- | --- | --- | --- | --- | --- | --- | --- | --- | --- | --- | --- | --- | --- | --- | --- | --- | --- | --- | --- | --- | --- | --- | --- | --- | --- | --- | --- | --- | --- | --- | --- | --- | --- | --- | --- | --- | --- | --- | --- | --- | --- | --- | --- | --- | --- | --- | --- | --- | --- | --- | --- | --- | --- | --- | --- | --- | --- | --- | --- | --- | --- | --- | --- | --- | --- | --- | --- | --- | --- | --- | --- | --- | --- | --- | --- | --- | --- | --- | --- | --- | --- | --- | --- | --- | --- | --- | --- | --- | --- | --- | --- | --- | --- | --- | --- | --- | --- | --- | --- | --- | --- | --- | --- | --- | --- | --- | --- | --- | --- | --- | --- | --- | --- | --- | --- | --- | --- | --- | --- | --- | --- | --- | --- | --- | --- | --- | --- | --- | --- | --- | --- | --- | --- | --- | --- | --- | --- | --- | --- | --- | --- | --- | --- | --- | --- | --- | --- | --- | --- | --- | --- | --- | --- | --- | --- | --- | --- | --- | --- | --- | --- | --- | --- | --- | --- | --- | --- | --- | --- | --- | --- | --- | --- | --- | --- | --- | --- | --- | --- | --- | --- | --- | --- | --- | --- | --- | --- | --- | --- | --- | --- | --- | --- | --- | --- | --- | --- | --- | --- | --- | --- | --- | --- | --- | --- | --- | --- | --- | --- | --- | --- | --- | --- | --- | --- | --- | --- | --- | --- | --- | --- | --- | --- | --- | --- | --- | --- | --- | --- | --- | --- | --- | --- | --- | --- | --- | --- | --- | --- | --- | --- | --- | --- | --- | --- | --- | --- | --- | --- | --- | --- | --- | --- | --- | --- | --- | --- | --- | --- | --- | --- | --- | --- | --- | --- | --- | --- | --- | --- | --- | --- | --- | --- | --- | --- | --- | --- | --- | --- | --- | --- | --- | --- | --- | --- | --- | --- | --- | --- | --- | --- | --- | --- | --- | --- | --- | --- | --- | --- | --- | --- | --- | --- | --- | --- | --- | --- | --- | --- | --- | --- | --- | --- | --- | --- | --- | --- | --- | --- | --- | --- | --- | --- | --- | --- | --- | --- | --- | --- | --- | --- | --- | --- | --- | --- | --- | --- | --- | --- | --- | --- | --- | --- | --- | --- | --- | --- | --- | --- | --- | --- | --- | --- | --- | --- | --- | --- | --- | --- | --- | --- | --- | --- | --- | --- | --- | --- | --- | --- | --- | --- | --- | --- | --- | --- | --- | --- | --- | --- | --- | --- | --- | --- | --- | --- | --- | --- | --- | --- | --- | --- | --- | --- | --- | --- | --- | --- | --- | --- | --- | --- | --- | --- | --- | --- | --- | --- | --- | --- | --- | --- | --- | --- | --- | --- | --- | --- | --- | --- | --- | --- | --- | --- | --- | --- | --- | --- | --- | --- | --- | --- | --- | --- | --- | --- | --- | --- | --- | --- | --- | --- | --- | --- | --- | --- | --- | --- | --- | --- | --- | --- | --- | --- | --- | --- | --- | --- | --- | --- | --- | --- | --- | --- | --- | --- | --- | --- | --- | --- | --- | --- | --- | --- | --- | --- | --- | --- | --- | --- | --- | --- | --- | --- | --- | --- | --- | --- | --- | --- | --- | --- | --- | --- | --- | --- | --- | --- | --- | --- | --- | --- | --- | --- | --- | --- | --- | --- | --- | --- | --- | --- | --- | --- | --- | --- | --- | --- | --- | --- | --- | --- | --- | --- | --- | --- | --- | --- | --- | --- | --- | --- | --- | --- | --- | --- | --- | --- | --- | --- | --- | --- | --- | --- | --- | --- | --- | --- | --- | --- | --- | --- | --- | --- | --- | --- | --- | --- | --- | --- | --- | --- | --- | --- | --- | --- | --- | --- | --- | --- | --- | --- | --- | --- | --- | --- | --- | --- | --- | --- | --- | --- | --- | --- | --- | --- | --- | --- | --- | --- | --- | --- | --- | --- | --- | --- | --- | --- | --- | --- | --- | --- | --- | --- | --- | --- | --- | --- | --- | --- | --- | --- | --- | --- | --- | --- | --- | --- | --- | --- | --- | --- | --- | --- | --- | --- | --- | --- | --- | --- | --- | --- | --- | --- | --- | --- | --- | --- | --- | --- | --- | --- | --- | --- | --- | --- | --- | --- | --- | --- | --- | --- | --- | --- | --- | --- | --- | --- | --- | --- | --- | --- | --- | --- | --- | --- | --- | --- | --- | --- | --- | --- | --- | --- | --- | --- | --- | --- | --- | --- | --- | --- | --- | --- | --- | --- | --- | --- | --- | --- | --- | --- | --- | --- | --- | --- | --- | --- | --- | --- | --- | --- | --- | --- | --- | --- | --- | --- | --- | --- | --- | --- | --- | --- | --- | --- | --- | --- | --- | --- | --- | --- | --- | --- | --- | --- | --- | --- | --- | --- | --- | --- | --- | --- | --- | --- | --- | --- | --- | --- | --- | --- | --- | --- | --- | --- | --- | --- | --- | --- | --- | --- | --- | --- | --- | --- | --- | --- | --- | --- | --- | --- | --- | --- | --- | --- | --- | --- | --- | --- | --- | --- | --- | --- | --- | --- | --- | --- | --- | --- | --- | --- | --- | --- | --- | --- | --- | --- | --- | --- | --- | --- | --- | --- | --- | --- | --- | --- | --- | --- | --- | --- | --- | --- | --- | --- | --- | --- | --- | --- | --- | --- | --- | --- | --- | --- | --- | --- | --- | --- | --- | --- | --- | --- | --- | --- | --- | --- | --- | --- | --- | --- | --- | --- | --- | --- | --- | --- | --- | --- | --- | --- | --- | --- | --- | --- | --- | --- | --- | --- | --- | --- | --- | --- | --- | --- | --- | --- | --- | --- | --- | --- | --- | --- | --- | --- | --- | --- | --- | --- | --- | --- | --- | --- | --- | --- | --- | --- | --- | --- | --- | --- | --- | --- | --- | --- | --- | --- | --- | --- | --- | --- | --- | --- | --- | --- | --- | --- | --- | --- | --- | --- | --- | --- | --- | --- | --- | --- | --- | --- | --- | --- | --- | --- | --- | --- | --- | --- | --- | --- | --- | --- | --- | --- | --- | --- | --- | --- | --- | --- | --- | --- | --- | --- | --- | --- | --- | --- | --- | --- | --- | --- | --- | --- | --- | --- | --- | --- | --- | --- | --- | --- | --- | --- | --- | --- | --- | --- | --- | --- | --- | --- | --- | --- | --- | --- | --- | --- | --- | --- | --- | --- | --- | --- | --- | --- | --- | --- | --- | --- | --- | --- | --- | --- | --- | --- | --- | --- | --- | --- | --- | --- | --- | --- | --- | --- | --- | --- | --- | --- | --- | --- | --- | --- | --- | --- | --- | --- | --- | --- | --- | --- | --- | --- | --- | --- | --- | --- | --- | --- | --- | --- | --- | --- | --- | --- | --- | --- | --- | --- | --- | --- | --- | --- | --- | --- | --- | --- | --- | --- | --- | --- | --- | --- | --- | --- | --- | --- | --- | --- | --- | --- | --- | --- | --- | --- | --- | --- | --- | --- | --- | --- | --- | --- | --- | --- | --- | --- | --- | --- | --- | --- | --- | --- | --- | --- | --- | --- | --- | --- | --- | --- | --- | --- | --- | --- | --- | --- | --- | --- | --- | --- | --- | --- | --- | --- | --- | --- | --- | --- | --- | --- | --- | --- | --- | --- | --- | --- | --- | --- | --- | --- | --- | --- | --- | --- | --- | --- | --- | --- | --- | --- | --- | --- | --- | --- | --- | --- | --- | --- | --- | --- | --- | --- | --- | --- | --- | --- | --- | --- | --- | --- | --- | --- | --- | --- | --- | --- | --- | --- | --- | --- | --- | --- | --- | --- | --- | --- | --- | --- | --- | --- | --- | --- | --- | --- | --- | --- | --- | --- | --- | --- | --- | --- | --- | --- | --- | --- | --- | --- | --- | --- | --- | --- | --- | --- | --- | --- | --- | --- | --- | --- | --- | --- | --- | --- | --- | --- | --- | --- | --- | --- | --- | --- | --- | --- | --- | --- | --- | --- | --- | --- | --- | --- | --- | --- | --- | --- | --- | --- | --- | --- | --- | --- | --- | --- | --- | --- | --- | --- | --- | --- | --- | --- | --- | --- | --- | --- | --- | --- | --- | --- | --- | --- | --- | --- | --- | --- | --- | --- | --- | --- | --- | --- | --- | --- | --- | --- | --- | --- | --- | --- | --- | --- | --- | --- | --- | --- | --- | --- | --- | --- | --- | --- | --- | --- | --- | --- | --- | --- | --- | --- | --- | --- | --- | --- | --- | --- | --- | --- | --- | --- | --- | --- | --- | --- | --- | --- | --- | --- | --- | --- | --- | --- | --- | --- | --- | --- | --- | --- | --- | --- | --- | --- | --- | --- | --- | --- | --- | --- | --- |
